# Post‐hospitalization rehabilitation alleviates long‐term immune repertoire alteration in COVID‐19 convalescent patients

**DOI:** 10.1111/cpr.13450

**Published:** 2023-03-20

**Authors:** Bing Feng, Danwen Zheng, Laijun Yang, Zuqing Su, Lipeng Tang, Ying Zhu, Xiaohua Xu, Qian Wang, Qiaoli Lin, Jiajun Hu, Meixuan Lin, Liqun Huang, Xin Zhou, Han Liu, Song Li, Wenjing Pan, Rongdong Shi, Yanjing Lu, Bin Wu, Banghan Ding, Zhe Wang, Jianwen Guo, Zhongde Zhang, Guangjuan Zheng, Yuntao Liu

**Affiliations:** ^1^ Guangdong Provincial Hospital of Chinese Medicine & Guangdong Provincial Academy of Chinese Medical Sciences Guangzhou China; ^2^ Guangdong‐Hong Kong‐Macau Joint Lab on Chinese Medicine and Immune Disease Research Guangzhou University of Chinese Medicine Guangzhou China; ^3^ State Key Laboratory of Dampness Syndrome of Chinese Medicine The Second Affiliated Hospital of Guangzhou University of Chinese Medicine Guangzhou China; ^4^ Guangdong Provincial Key Laboratory of Chinese Medicine for Prevention and Treatment of Refractory Chronic Diseases Guangzhou China; ^5^ The Second Clinical College of Guangzhou University of Chinese Medicine Guangzhou China; ^6^ Hunan Key Laboratory of Biomedical Nanomaterials and Devices Hunan University of Technology Zhuzhou China; ^7^ Hengyang Medical School University of South China Hengyang China

## Abstract

The global pandemic of Coronavirus disease 2019 (COVID‐19) caused by severe acute respiratory syndrome coronavirus 2 (SARS‐CoV‐2) is an once‐in‐a‐lifetime public health crisis. Among hundreds of millions of people who have contracted with or are being infected with COVID‐19, the question of whether COVID‐19 infection may cause long‐term health concern, even being completely recovered from the disease clinically, especially immune system damage, needs to be addressed. Here, we performed seven‐chain adaptome immune repertoire analyses on convalescent COVID‐19 patients who have been discharged from hospitals for at least 6 months. Surprisingly, we discovered lymphopenia, reduced number of unique CDR3s, and reduced diversity of the TCR/BCR immune repertoire in convalescent COVID‐19 patients. In addition, the BCR repertoire appears to be activated, which is consistent with the protective antibody titres, but serological experiments reveal significantly lower IL‐4 and IL‐7 levels in convalescent patients compared to those in healthy controls. Finally, in comparison with convalescent patients who did not receive post‐hospitalization rehabilitation, the convalescent patients who received post‐hospitalization rehabilitation had attenuated immune repertoire abnormality, almost back to the level of healthy control, despite no detectable clinic demographic difference. Overall, we report the potential long‐term immunological impairment for COVID‐19 infection, and correction of this impairment via post‐hospitalization rehabilitation may offer a new prospect for COVID‐19 recovery strategy.

## Graphical abstract

- SARS‐CoV‐2 infection induced potential long‐term immunological impairment.
- Holistic immune repertoire diversity decreased in convalescent COVID‐19 patients.
- Rehabilitation can normalize the impaired immune functions in convalescent COVID‐19 patients.

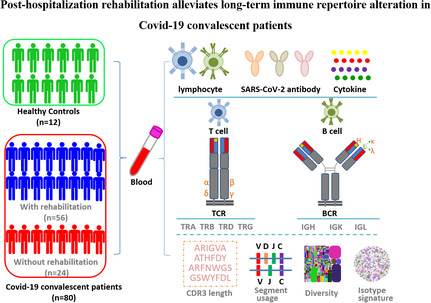

## 1 INTRODUCTION

The global pandemic of severe acute respiratory syndrome coronavirus 2 (SARS‐CoV‐2) is the most severe public health emergency event for decades.^1^ As of January 2023, SARS‐CoV‐2 had infected more than 660 million people and caused more than 6 million deaths worldwide. Although the epidemiological and clinical features, pathogenesis, and complications of patients infected with SARS‐CoV‐2 in the acute phase have been well explored and documented, studies on the clinical and scientific features of convalescent COVID‐19 patients remain limited.^2^, ^3^ Tremendous efforts have been made to develop therapeutic strategies and vaccines for COVID‐19 infection, yet the efficacy and specificity of various treatments needs to be further evaluated and tested.^4^ In addition, the long‐term effects of COVID‐19 infection on the immune system of convalescent COVID‐19 patients have been neglected. Therefore, it is an urgent need to explore the response of the immune system to SARS‐CoV‐2 infection in convalescent COVID‐19 patients. Moreover, evaluating and understanding the immune system change in convalescent COVID‐19 patients will also have great clinical, scientific and sociological significance.

Adaptive immunity confers a major immune response to viral infection,^5^ including COVID‐19 infection.^6^, ^7^ The adaptive immune system is mainly comprised of lymphocytes, of which T cells and B cells execute their cellular and humoral immune responses, respectively, via specific binding of pathogens with T cell receptor (TCR) and B cell receptor (BCR)^8^, ^9^ and the aggregates of the entire TCR/BCR repertoire termed adaptome.^10^, ^11^ Each TCR/BCR gene locus comprise variable (V), diversity (D), juncture (J) and constant (C) segments, of which V, D and J regions undergo genomic rearrangement and random nucleotide additions/deletions, resulting in a unique TCR and BCR for each T cell and B cell.^12^, ^13^, ^14^ The CDR3 (complementarity‐determining region 3) region is located at the junction of VDJ genes and provides the basis of immune repertoire diversity and antigen‐binding specificity of TCR and BCR.^13^, ^15^, ^16^ This random VDJ recombination bestows the extraordinary diversity of TCR/BCR immune repertoire, ranging from 10^15^ to 10^25^ for each chain, that is IgH (all isotypes), IgK, IgL, TCR‐Alpha, Beta chains, and TCR‐Gamma, Delta chains.^17^ The TCR and BCR diversity provides extensive protection against various pathogens and viruses.^18^

Various studies have profiled the TCR^19^ and BCR^20^, ^21^ immune repertoire separately and holistically^22^, ^23^ on COVID‐19 patients. Consistently, the TCR/BCR diversity is significantly decreased during the acute stage of COVID‐19 infection^19^, ^24^ in different disease cohorts. In addition, certain dynamic V/J preference, IgM to IgG class switch, B cell hypermutation, and clonotype expansion were also reported,^25^, ^26^, ^27^ describing the characteristic pattern of COVID‐19 infection.

As estimated, hundreds of millions, even billions of people have or will be infected with COVID‐19, it is of great importance to evaluate the clinical and immunologic characteristics of convalescent patients. Serologic analysis discovered that protective adaptive immune responses of anti‐SARS‐CoV‐2 antibodies and specific memory B and T cell responses caused by natural SARS‐CoV‐2 infection may last for at least 6–8 months after the onset of symptoms,^28^ and the diversity of the immune repertoire in convalescent patients with COVID‐19 is associated with outcomes.^29^

Although for most of the people infected with COVID‐19, the symptoms are mild, even asymptomatic,^30^, ^31^ the long‐term effect of COVID‐19 infection on the immune system still attracts tremendous public attention. Here, we discovered characteristic pan‐immune repertoire alterations, like lymphopenia, reduced unique CDR3 number and reduced diversity, together with the imbalance of V/J usage even in convalescent patients at least 6 months after hospitalization, indicating long‐term immune system marks upon COVID‐19 infection. Interestingly, convalescent patients with severe symptoms and underwent post‐hospitalization rehabilitation, have markedly improved immune repertoire profile compared to the convalescent patients without rehabilitation, justifying the necessity of rehabilitation upon COVID‐19 recovery.

## 2 MATERIALS AND METHODS

### 2.1 Sample collection

Screening of COVID‐19 convalescent patient donors and sample collection were conducted at Hubei Provincial Hospital of Traditional Chinese & Western Medicine. The study protocols were approved by the Ethical Committee of Guangdong Provincial Hospital of Chinese Medicine (No. BF2020‐205‐01), and all associated procedures were carried out in accordance with approved guidelines. All participants provided written informed consent in accordance with the Declaration of Helsinki. Eighty COVID‐19 convalescent patients (age > 18 years old) were enrolled in our study, who were hospitalized in Wuhan from January to April 2020. The median number of days from hospital discharge to return visit was 241.79 ± 16.16 days, ranging from 182 days to 293 days. Seven healthy volunteers were Wuhan citizens and five healthy volunteers were Guangzhou citizens, and they all lived in their city during the epidemic period. All 12 healthy volunteers confirmed that they were not infected with SARS‐CoV‐2. None of the subjects took COVID vaccine.

### 2.2 Sample collection

Blood samples were collected from patients with informed consent. Whole blood samples were collected via venipuncture and the sera were separated by centrifugation at 3000 × *g* for 15 min within 8 h after collection, and then the supernatant was collected. The sera were inactivated at 56°C for 30 min, and then stored at −80°C.

The peripheral blood samples for the immune repertoire sequence were collected via venipuncture using the PAXgene Blood RNA Tube. Then the samples were placed at room temperature for 4 h, and then transferred to −80°C until total RNA was extracted.

### 2.3 Cytokine and chemokine measurement

Cytokine and chemokine were measured using the Human Cytokine HSTCMAG‐28SK‐21 and TGFBMAG‐64K‐03 Assays panels, and the MILLIPLEX® MAGPIX system (Thermo Fisher, USA) according to the manufacturer's instructions.

### 2.4 Anti‐SARS‐CoV‐2 IgA, IgM, IgG and neutralizing antibody test

The IgA, IgM, IgG and neutralizing antibodies were tested by fully automated chemiluminescent immunoassays (AHLO, Shenzhen), and the cut‐off point was set to 10 AU/L. If the sample concentration is greater than or equal to 10 AU/L, it is considered reactive to SARS‐CoV‐2 IgA, IgM, IgG or neutralizing antibodies.

### 2.5 Detection of immune repertoire of T cell and B cell

Extracted total RNA was amplified using a commercially available iR‐RepSeq‐plus 7‐Chain Cassette (iRepertoire Inc, US) covering the human TCR and BCR all seven chains using a strategy that allows the incorporation of unique molecular identifiers during the reverse transcription (RT) step. One disposable cassette is for one sample's library preparation; all necessary reagents for amplification and purification are preloaded into the cassette. For the construction of human TCR and BCR libraries, RT is performed using Qiagen OneStep RT PCR mix (Qiagen). First‐strand cDNA is selected and unused primer is removed by SPRIselect bead selection (Beckman Coulter), followed by the second round of binding and extension with the V‐gene primer mix. After binding and extension, SPRIselect beads are used to purify the first and second strand synthesis products. Library amplification is performed with a pair of primers that are specific for communal sites engineered onto the 5′ end of the C‐ and V‐primers used in the first and second strand synthesis. The final constructed library includes Illumina dual index sequencing adapters, a 10‐nucleotide unique molecular identifier region, and an 8‐nucleotide internal barcode associated with the C‐gene primer.

Amplified libraries were multiplexed and pooled for sequencing on the Illumina MiSeq platform using a 600‐cycle kit and were sequenced as 250 paired‐end reads. The output of the immune receptor sequence covers the second framework region through the beginning of the constant region including CDR2 and CDR3. Sequencing raw data were analysed using the previously described iRmap program (Wang et al., PNAS, 2010; Yang et al., Elife, 2015). Briefly, sequence reads were de‐multiplexed according to both Illumina dual indices incorporated during the amplification process and barcode sequences at the 5′ end of reads from the constant region. Reads were then trimmed according to their base qualities with a 2‐base sliding window. If either quality value in this window is lower than 20, this sequence stretches from the window to 3′ end is trimmed out from the original read. Trimmed pair‐end reads were joined together through overlapping alignment with a modified Needleman‐Wunsch algorithm. If paired forward and reverse reads in the overlapping region were not perfectly matched, both forward and reverse reads were thrown out without further consideration. The merged reads were mapped using a Smith‐Waterman algorithm to germline V, D, J and C reference sequences using an IMGT reference library. To define the complementary determining regions three (CDR3s) region, the position of CDR3 boundaries of reference sequences from the IMGT database was migrated onto reads through mapping results, and the resulting CDR3 regions were extracted and translated into amino acids.

### 2.6 Statistical analyses

Statistical analyses were performed using SPSS version 20 (SPSS, Chicago, IL). Statistical significance between two groups was determined using Mann–Whitney *U* test, and between multiple groups by Kruskal–Wallis test.

## 3 RESULTS

### 3.1 Altered adaptome in convalescent COVID‐19 patients

To discover the potential long‐term effect of COVID‐19 infection on convalescent patients, we collected 80 peripheral blood mononuclear cell (PBMC) samples from convalescent COVID‐19 patients and 12 healthy donors as normal control. The demographic and clinical characteristics of the patients are shown in Table 1. In the convalescent group, 30 (37.5%) are male and 50 (62.5%) are female, and in the control group, 6 (50%) are male and 6 (50%) are female. Among convalescent patients, 63 (78.7%) had a mild/moderate disease score and 17 (21.3%) had a severe/critical disease score during hospitalization with SARS‐CoV‐2 infection. Of the 80 total convalescent patients, 36 (45%) had one or more basic diseases, such as hypertension (22.5%), diabetes (16.25%), heart diseases (3.75%), liver disease (2.5%), lung diseases (3.75%), cancer (5%) and chronic obstructive pulmonary disease (2.5%).

**TABLE 1:** Summary of demographic and clinical characteristics in convalescent COVID‐19 individuals and healthy individuals.

	Convalescent COVID‐19 individuals	Healthy individuals
Demographics		
Number	80	12
Men, %	37.5% (30/80)	50% (6/12)
Women, %	62.5% (50/80)	50% (6/12)
Age, y, median (range)	60 (34–87)	55 (22–67)
SARS‐CoV‐2 antibody		
IgA (negative/positive)	37/43	12/0
IgM (negative/positive)	60/20	12/0
IgG (negative/positive)	6/74	12/0
Neutralizing antibodies (negative/positive)	6/74	12/0
Medical history, %		
Hypertension	22.5% (18/80)	16.7% (2/12)
Diabetes	16.25% (13/80)	8.3% (1/12)
Heart diseases	3.75% (3/80)	0% (0/12)
Liver disease	2.5% (2/80)	0% (0/12)
Lung diseases	3.75% (3/80)	8.3% (1/12)
Tumour	5% (4/80)	8.3% (1/12)
Rheumatism	2.5% (2/80)	0% (0/12)
Other comorbidities	5% (4/80)	8.3% (1/12)
>1 comorbidity	8.75% (7/80)	16.7% (2/12)
>2 comorbidities	2.5% (2/80)	8.3% (1/12)

**FIGURE 1:**
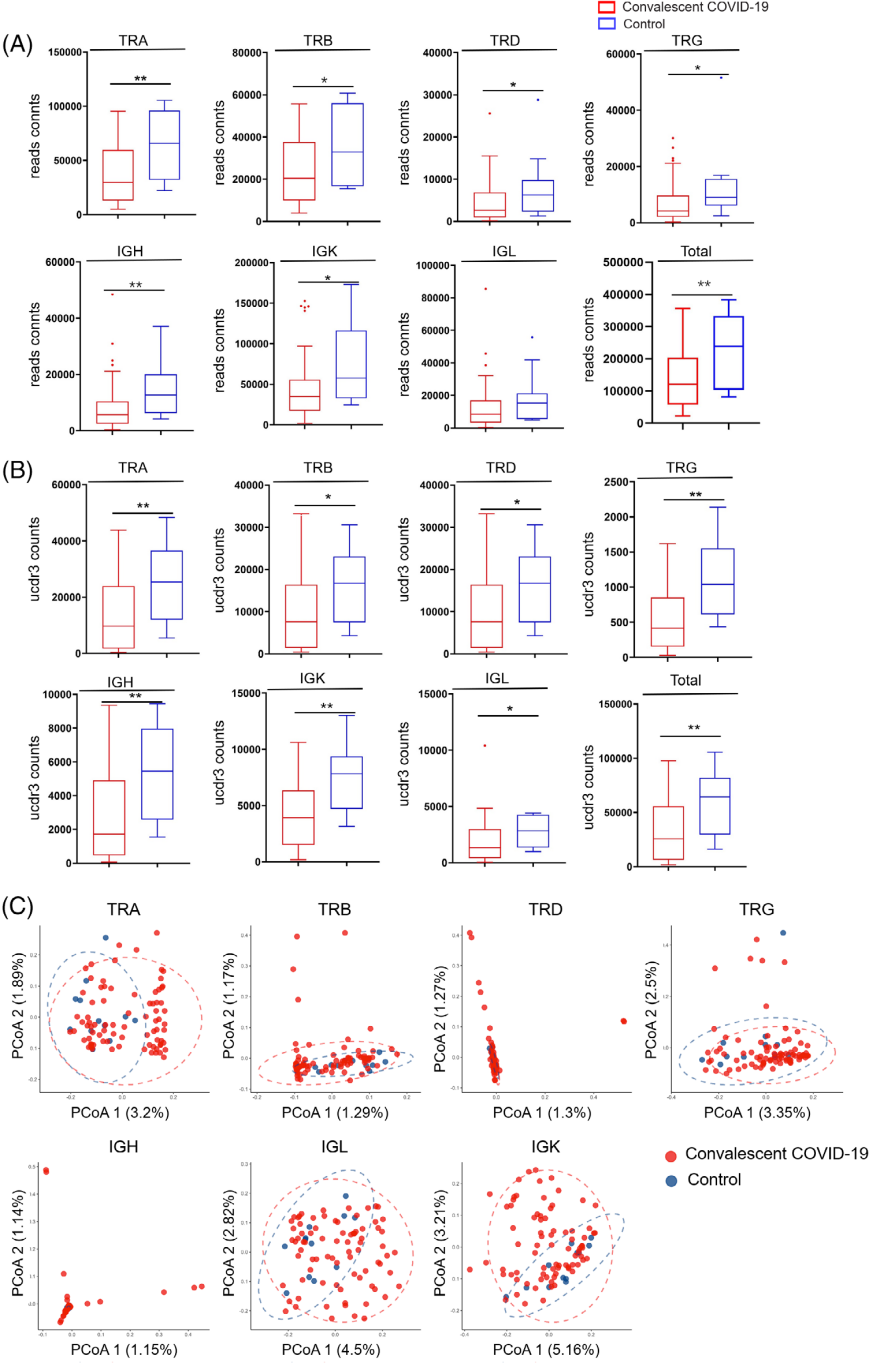
Adaptome profiling, that is immune repertoires in PBMCs of convalescent COVID‐19 patients. The proportion of reads for overall adaptome immune repertoire and seven‐chain adaptome immune repertoire (A), are presented as mean value ± SD in the boxplots. The box extends from the 25th to 75th percentiles. The bars go down to the smallest value and up to the largest value. The abundance of immune repertoire measured by the number of unique CDR3 (uCDR3) in the overall adaptome and seven‐chain adaptome (B). Principle of component analysis (PoCA) based on the abundance of TRA, TRB, TRD, TRG, IGH, IGK and IGL clones and the distance between the dots indicates the degree of dissimilarity between samples (C). *p < 0.05, **p < 0.01.

Various studies have reported lymphopenia in COVID‐19 patients, represented by diminished lymphocyte^32^, ^33^ number and reduced expression of TCR and BCR.^19^ For convalescent patients, who were discharged from the hospital for at least 180 days, unique TCR reads remained significantly lower than healthy controls (Figure 1A), which is consistent with the results of the FACS analysis (Figure 5A). Adaptome immune repertoire consists of TCR chains (TCR‐alpha, TRA; TCR‐beta, TRB; TCR‐delta, TRD; TCR‐gamma, TRG) and BCR chains (IgH, including various IgH isotypes; IgK; and IgL). Within the repertoire of each chain, compared to healthy controls, reads counts of convalescent patients reduced in TRA (35,932 vs. 62,941), TRB (23,977 vs. 35,257), TRD (4329 vs. 7969), TRG (7282 vs. 13,203), IgH (7817 vs. 9242), IgK (42,853 vs. 72,472) and IgL (12,309 vs. 18,019) .

A unique CDR3 (uCDR3) sequence is also defined as a clonotype, which targets a particular antigen, therefore, the uCDR3 may reflect the total adaptive immunity.^34^, ^35^ Compared to healthy control, convalescent patients displayed a reduced uCDR3 count (Figure 1B), of which the average uCDR3 count for each chain was all reduced.

Principal coordinate analysis (PCoA) was performed on the uCDR3 frequency profile of the adaptome immune repertoire. Significant differences were discovered between healthy controls and convalescent patient groups in TCR and BCR clusters were separated in PCoA plots (Figure 1C).

Taken together, we discovered reduced lymphocyte numbers as well as unique CDR3 numbers in the convalescent patients, which may suggest impaired long‐term immune repertoire following COVID‐19 infection. Moreover, overall TCR and BCR features were also altered represented by PCoA.

### 3.2 Holistic immune repertoire diversity changed in convalescent COVID‐19 patients

**FIGURE 2:**
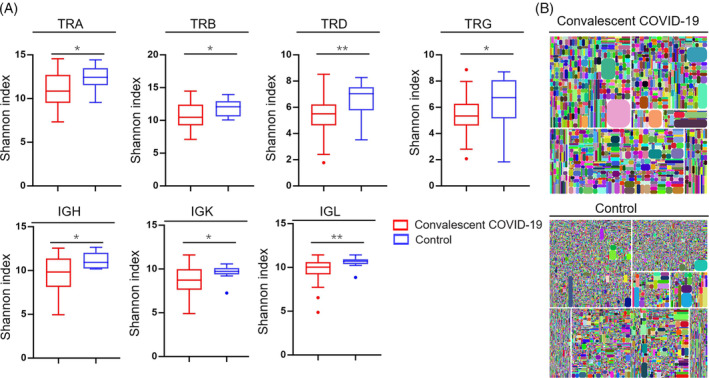
The diversity of adaptome in PBMCs of convalescent COVID‐19 patients. The diversity of was demonstrated shannon‐index at the level of unique uCDR3 clones, as shown of TRA, TRB, TCD, TRG, IgH, IgK and IgL (A). Immune repertoire diversity is shown by tree maps in two representative individual samples (B), in which each round rectangle represents a unique uCDR3, and the size of each rectangle denotes the relative frequency of that uCDR3 clone. *p < 0.05, **p < 0.01.

To further explore the potential long‐term immune repertoire damage caused by COVID‐19 contraction, the diversity of TCR and BCR repertoires was measured in all samples. Diversity, measured with Shannon entropy, describes the evenness of the immune repertoire. Compared to healthy control, convalescent patients showed a significant decrease in Shannon entropy for all seven chains of the immune repertoire, TCRA (10.99 vs. 12.35), TCRB (10.68 vs. 11.93), TCRD (5.38 vs. 6.32), TCRG (5.43 vs. 6.61), IgH (9.61 vs. 11.15), IgK (9.77 vs. 10.61) and IgL (8.72 vs. 9.64) (Figure 2).

In summary, we discovered the diminished TCR/BCR repertoire diversity in convalescent patients, further implying the long‐term effect of COVID‐19 infection on the adaptome.

### 3.3 Skewed TRBV/J and IGHV/J gene usage in convalescent COVID‐19 patients

**FIGURE 3:**
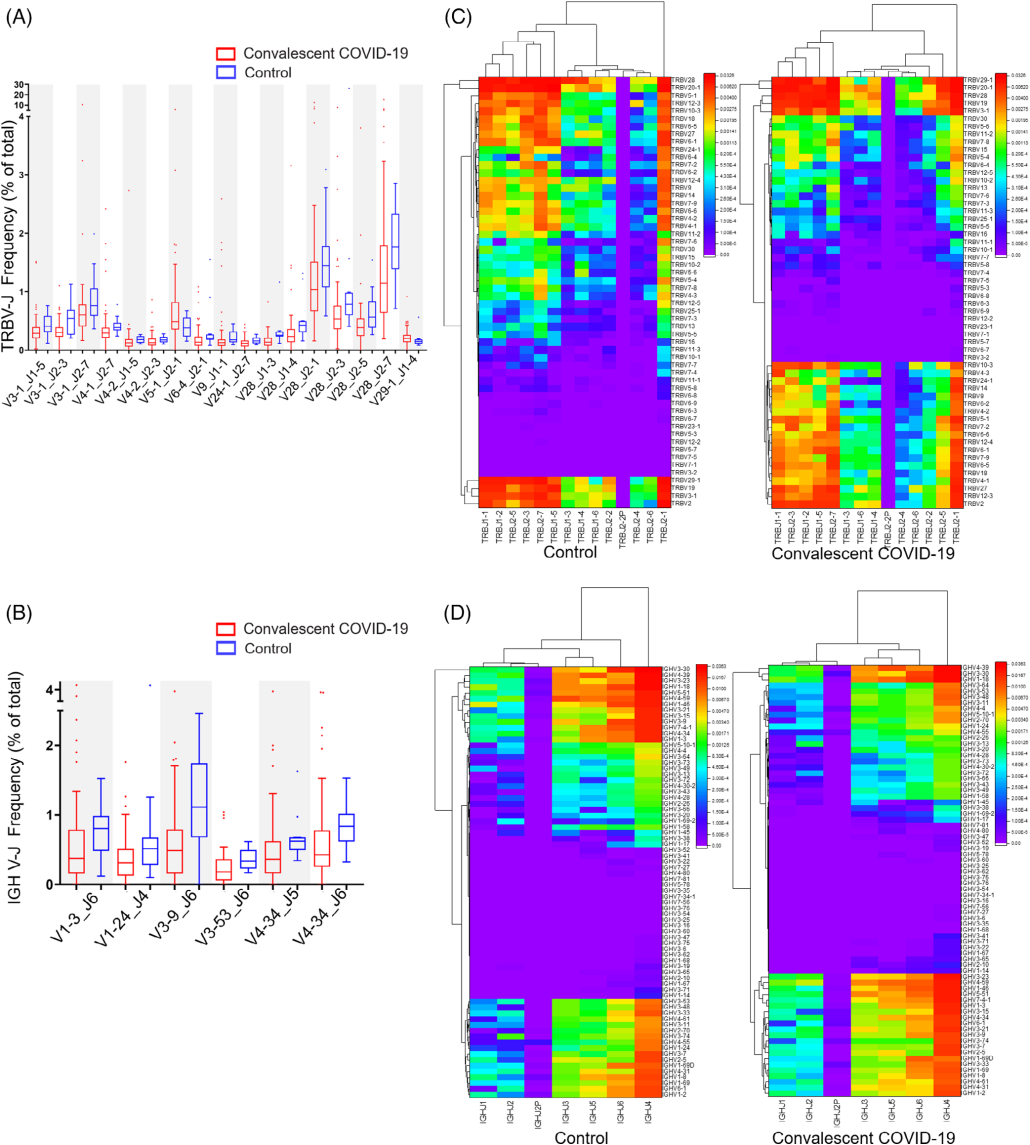
V/J preference usage of convalescent COVID‐19 patients. Mann–Whitney *U* test was used to statistically define the significant difference, and data are presented as the mean ± SD values. All of the TRBV‐TRBJs conjunctions (A) and IGHV‐IGHJ conjunctions (B) with *p* value <0.05, between control and convalescent COVID‐19 patients are presented. Heat maps of V gene usage in conjunction with J usage in convalescent COVID‐19 patients showing preferred TRBV–TRBJ pairs (C) and IGHV‐IGHJ pairs (D).

To further evaluate whether there is a preferential use of V and J genes in convalescent COVID‐19 patients, we calculated the frequencies of TRBV, TRBJ, IGHV and IGHJ in convalescent patients compared to healthy controls. Significant higher usage of TRBV7‐2 and TRBV11‐3, and lower usage of TRBV4‐2, TRBV20‐1, TRBV28, IGHV1‐58, IGHV3‐9 and IGHV4‐34 were discovered in convalescent patients compared to healthy controls (Figure S1). Moreover, TRBV5‐1_TRBJ2‐1 pair and TRBV29‐1_TRBJ1‐4 pair are preferably expressed in convalescent patients and numerous pairs including TRBV3‐1_TRBJ1‐5, TRBV3‐1_TRBJ2‐3, TRBV3‐1_TRBJ2‐7, TRBV4‐1_TRBJ2‐7, TRBV4‐2_TRBJ1‐5, TRBV4‐2_TRBJ2‐3, TRBV6‐4_TRBJ2‐1, TRBV9_TRBJ1‐1, TRBV24‐1_TRBJ2‐7, TRBV28_TRBJ1‐3, TRBV28_TRBJ1‐4, TRBV28_TRBJ2‐1, TRBV28_TRBJ2‐3, TRBV28_TRBJ2‐5, TRBV28_TRBJ2‐7 (Figure 3A), IGHV3‐1_IGHJ6, IGHV1‐24_IGHJ4, IGHV1‐24_IGHJ4, IGHV3‐53_IGHJ6, IGHV4‐34_IGHJ5, IGHV4‐34_IGHJ6 (Figure 3B) were reduced expressed in convalescent patients.

On the other hand, we discovered no substantial differences in CDR3 length and amino acid composition between healthy control and convalescent patients (Figure S2).

### 3.4 BCR repertoire display activation pattern

**FIGURE 4:**
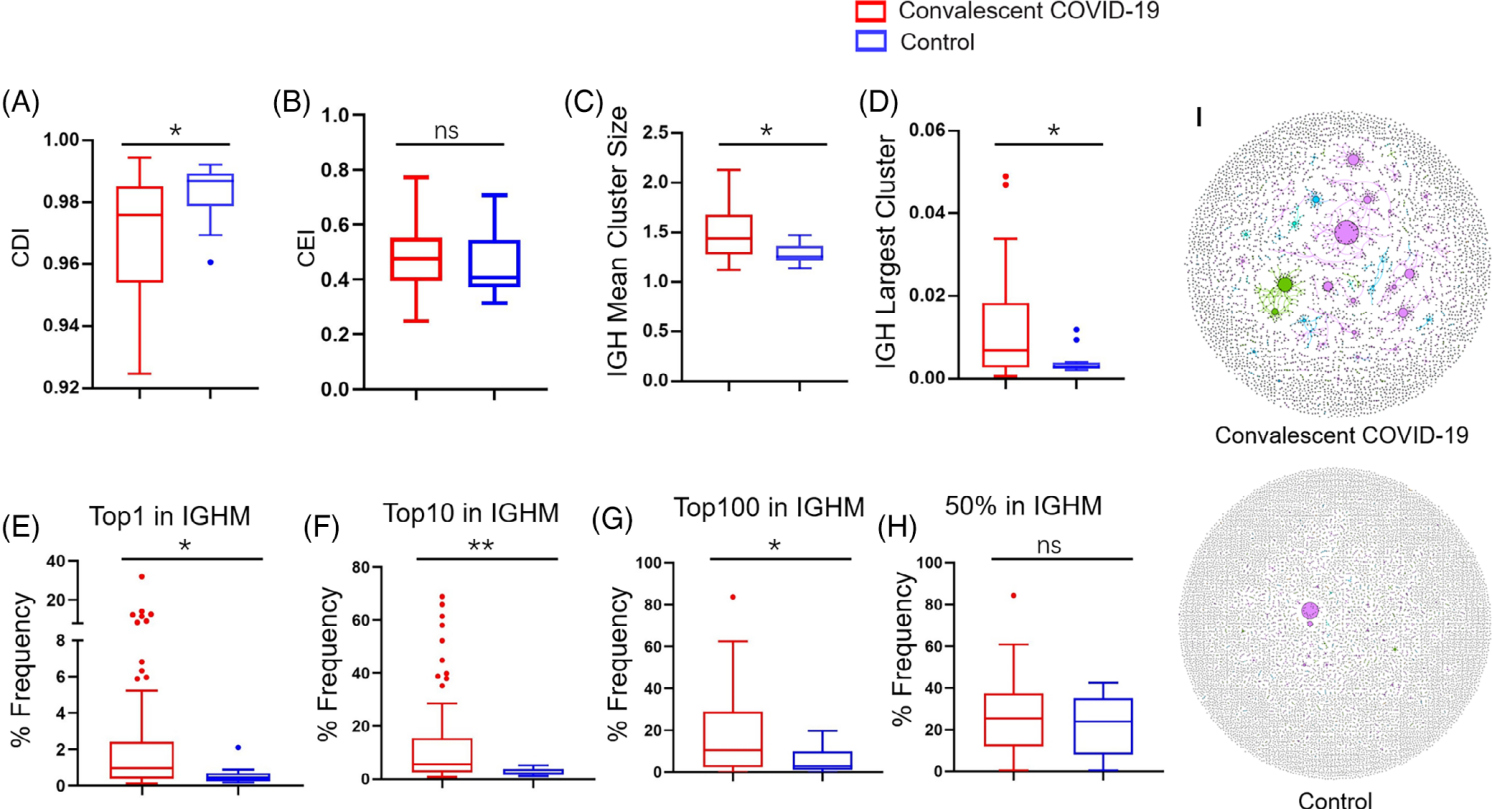
B cell repertoire clonality and clonal network parameters of convalescent COVID‐19 patients. Clonal expansion index (A) and clonal diversification index of BCRs (B) for BCR repertoires in PBMCs. Between healthy control and convalescent COVID‐19 patients, IGH mean cluster size (C) and IGH largest clusters (D) were shown, as well as the percentage expression of the largest clone for IGHM out of the total expression of all sequences belonging to IGHM (E), the percentage expression of the top 10 largest clones for IGHM out of the total expression of all sequences belonging to IGHM (F), the percentage of reads that for IGHM of the top 100 unique clones of the sample (G) and the percentage of reads for IGHM out of the top 50% of reads in the repertoire (H). B cell hypermutation is shown in two representative individual samples (I), in which each dot represents a unique B cell uCDR3, and the size of each dot denotes the relative frequency of that uCDR3 clone, and the dots connected by line represent hypermutation.

Previous study has described the characteristic dynamics of the B cell repertoire after COVID‐19 infection, yet the study scope was limited to 1 month after infection.^19^ Therefore, in order to follow up on the feature of the B‐cell repertoire over time, we further explored the B‐cell repertoire in our study cohort. Utilizing the algorithm described by Bashford‐rogers et al.,^36^ the B cell clones are measured by size (clonal expansion), which is characterized as clone expansion index (CEI), and diversification (somatic hypermutation and isotype switching), which is characterized as clone diversification index (CDI). CEI is used to indicate the unevenness of the number of RNA molecules per VDJ region sequences, which is defined by the Gini index, and CDI is used to indicate the unevenness of unique VDJ region sequences per clone, which is defined by Renyi entropy. In our study, clonal diversification (Figure 4A) was decreased in convalescent patients. This trend of clone diversification, a comprehensive measure of B cell clone diversity, hypermutation and isotype switching, was discovered in all IgH isotypes, including IgHA, IgHD, IgHE, IgHG and IgHM (Figure S3). Moreover, larger mean IgHM cluster sizes, largest IgHM clones (Figure 4C,D), larger mean IgHD cluster sizes, and largest IgHD clones were discovered in convalescent patients (Figures S3 and S4). Overall, we found the B cell repertoire remained activated in convalescent patients.

### 3.5 Serological analysis of convalescent COVID‐19 patients

**FIGURE 5:**
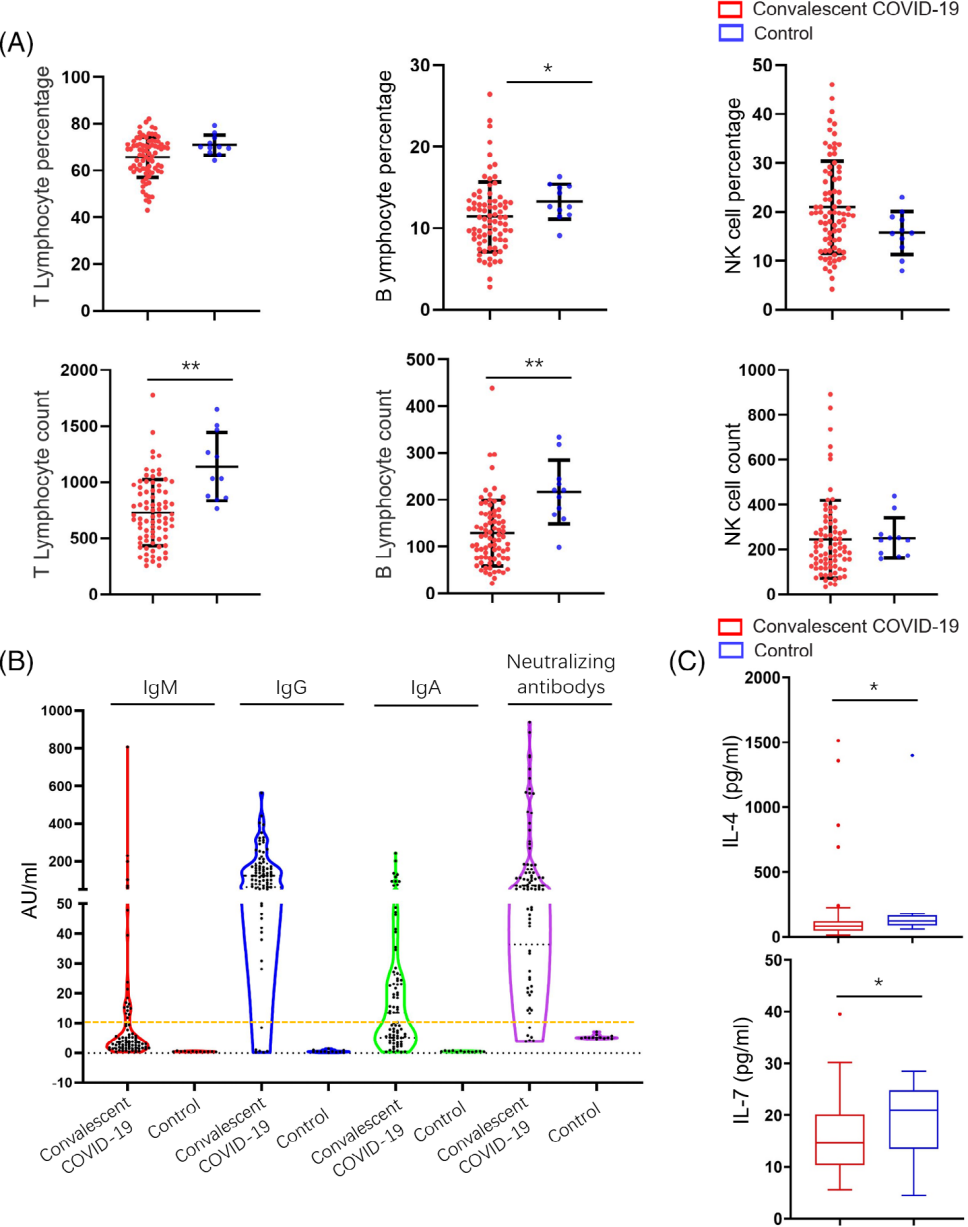
The lymphocyte, cytokines and SARS‐CoV‐2 specific immunoglobulin in peripheral blood of convalescent COVID‐19 patients. T cell, B cell and NK cell count were measured using flow cytometry (A). Levels of SARS‐CoV‐2 specific immunoglobulin isotypes of IgA, IgM, IgG and neutralizing antibody (B) were measured by ELISA, between control and convalescent COVID‐19 patients. Plasma cytokine IL‐4 and IL‐7 were measured using Luminex, resulting in the mean of plate‐detrended median fluorescence intensity values of each cytokine per sample (C). Mann–Whitney *U* test were performed for each cytokine, comparing healthy controls with convalescent COVID‐19 patients.

Cytokines produced by COVID‐19 infection play a crucial role in the aetiology and pathophysiology of the disease, especially the overproduction of pro‐inflammatory cytokines such as IL‐1, IL‐6, IL‐12, IFN‐γ and TNF‐α,^37^, ^38^, ^39^ and the levels of these cytokines correlate with disease prognosis.^40^, ^41^ However, the serological cytokine profile of convalescent COVID‐19 patients remains unclear. Therefore, we first performed immunoglobulin isotypes of IgA, IgM, IgG and neutralizing antibody analysis (Figure 5B). Moreover, we conducted cytokine profiling study on our serum samples (Figure 5C, Figure S5). Of the 24 cytokines measured, only IL‐4 and IL‐7 were significantly decreased in convalescent patients when compared to healthy controls. IL‐4 has anti‐inflammatory properties and elevated IL‐4 levels have been reported as part of a cytokine storm associated with severe respiratory symptoms^42^, ^43^; IL‐7 can activate T cells, and its levels are elevated in COVID‐19 patients, directly related to disease severity.^44^, ^45^ Here, our finding demonstrated the abnormal level of certain cytokines in convalescent COVID‐19 patients, indicating residual long‐term effect of the infection., and reduced IL‐7 level may correlate with diminished T cell number and function.

### 3.6 Post‐hospitalization rehabilitation enhances immune repertoire normalization in convalescent patients

Of 80 convalescent patients, 56 of them performed post‐hospitalization rehabilitation (PHR) after being discharged from hospitals (Table 2). Rehabilitation includes sports training, breath training, and traditional Chinese medicine intervention. etc. (Table 3). We found no significant difference in CT, respiratory function, and blood biochemical examination between the 56 PHR patients and the 24 non‐PHR patients (Table 2).

**TABLE 2:** Demographic and clinical characteristics of the convalescent COVID‐19 individuals.

		Non‐post‐hospitalization rehabilitation	Post‐hospitalization rehabilitation	*p* value
Sex	Male	7 (29.2%)	23 (41.1%)	0.45
	Female	17 (70.8%)	33 (58.9%)	
Age	Age, median	39–87, 57	34–80, 61	0.311
Disease severity	Mild/moderate	21 (87.5%)	42 (75%)	0.249
	Severe/critical	3 (12.5%)	14 (25%)	
Medical history	No	13 (54.2%)	31 (55.4%)	1
	Yes	11 (45.8)	25 (44.6)	
Hypertension	No	20 (83.3%)	42 (75%)	0.562
	Yes	4 (16.7%)	14 (25%)	
Diabetes	No	19 (79.2%)	48 (85.7%)	0.692
	Yes	5 (20.8%)	8 (14.3%)	
Heart diseases	No	22 (91.7%)	55 (98.2%)	0.213
	Yes	2 (8.3%)	1 (1.8%)	
Liver disease	No	23 (95.8%)	55 (98.2%)	0.513
	Yes	1 (4.2%)	1 (1.85%)	
Lung diseases	No	23 (95.8%)	54 (96.4%)	1
	Yes	1 (4.2%)	2 (3.6%)	
Tumour	No	23 (95.8%)	53 (94.6%)	1
	Yes	1 (4.2%)	3 (5.4%)	
Rheumatism	No	23 (95.8%)	55 (98.2%)	0.513
	Yes	1 (4.2%)	1 (1.85%)	

**TABLE 3:** Sequelae symptom of the convalescent COVID‐19 individuals.

		Non‐post‐hospitalization rehabilitation	Post‐hospitalization rehabilitation	*p* value
Abnormal pulmonary function	No	7 (53.8%)	14 (43.8%)	0.743
	Yes	6 (46.2%)	18 (56.2%)	
Ventilatory dysfunction	No	12 (92.3%)	30 (93.8%)	1
	Yes	1 (7.7%)	2 (6.2%)	
Small airway dysfunction	No	12 (92.3%)	24 (75%)	0.249
	Yes	1 (7.7%)	8 (25%)	
Diffusion dysfunction	No	9 (69.2%)	21 (65.6%)	1
	Yes	4 (30.8%)	11 (34.4%)	
Decreased exercise tolerance	No	16 (66.7%)	38 (69.1%)	1
	Yes	8 (33.3%)	17 (30.9%)	
PDST	No	23 (95.8%)	50 (89.3%)	0.604
	Yes	1 (4.2%)	6 (10.7%)	
Anxious	No	22 (91.7%)	51 (91.1%)	1
	Yes	2 (8.3%)	5 (8.9%)	
Fatigue	No	13 (54.2%)	24 (42.9%)	0.464
	Yes	11 (45.8%)	32 (57.1%)	
Shortness of breath	No	20 (83.3%)	41 (73.2%)	0.401
	Yes	4 (16.7%)	15 (26.8%)	
Spontaneous sweating	No	22 (91.7%)	41 (73.2%)	0.079
	Yes	2 (8.3%)	15 (26.8%)	
Muscle and joint pain	No	23 (95.8%)	51 (91.1%)	0.781
	Yes	1 (4.2%)	5 (8.9%)	
Aversion to cold	No	21 (87.5%)	46 (82.1%)	0.791
	Yes	3 (12.5%)	10 (17.9%)	
Chest tightness	No	21 (87.5%)	36 (64.3%)	0.057
	Yes	3 (12.5%)	20 (35.7%)	
Cough	No	23 (95.8%)	50 (89.3%)	0.604
	Yes	1 (4.2%)	6 (10.7%)	
Decreased appetite	No	21 (87.5%)	52 (92.9%)	0.73
	Yes	3 (12.5%)	4 (7.1%)	
Memory decline	No	23 (95.8%)	51 (91.1%)	0.781
	Yes	1 (4.2%)	5 (8.9%)	
Insomnia	No	19 (79.2%)	40 (71.4%)	0.585
	Yes	5 (20.8%)	16 (28.6%)	
Chest CT	Normal	6 (25%)	24 (42.9%)	0.077
	Acute inflammation	8 (33.3%)	7 (12.5%)	
	Chronic inflammation	10 (41.7%)	25 (44.6)	

**FIGURE 6:**
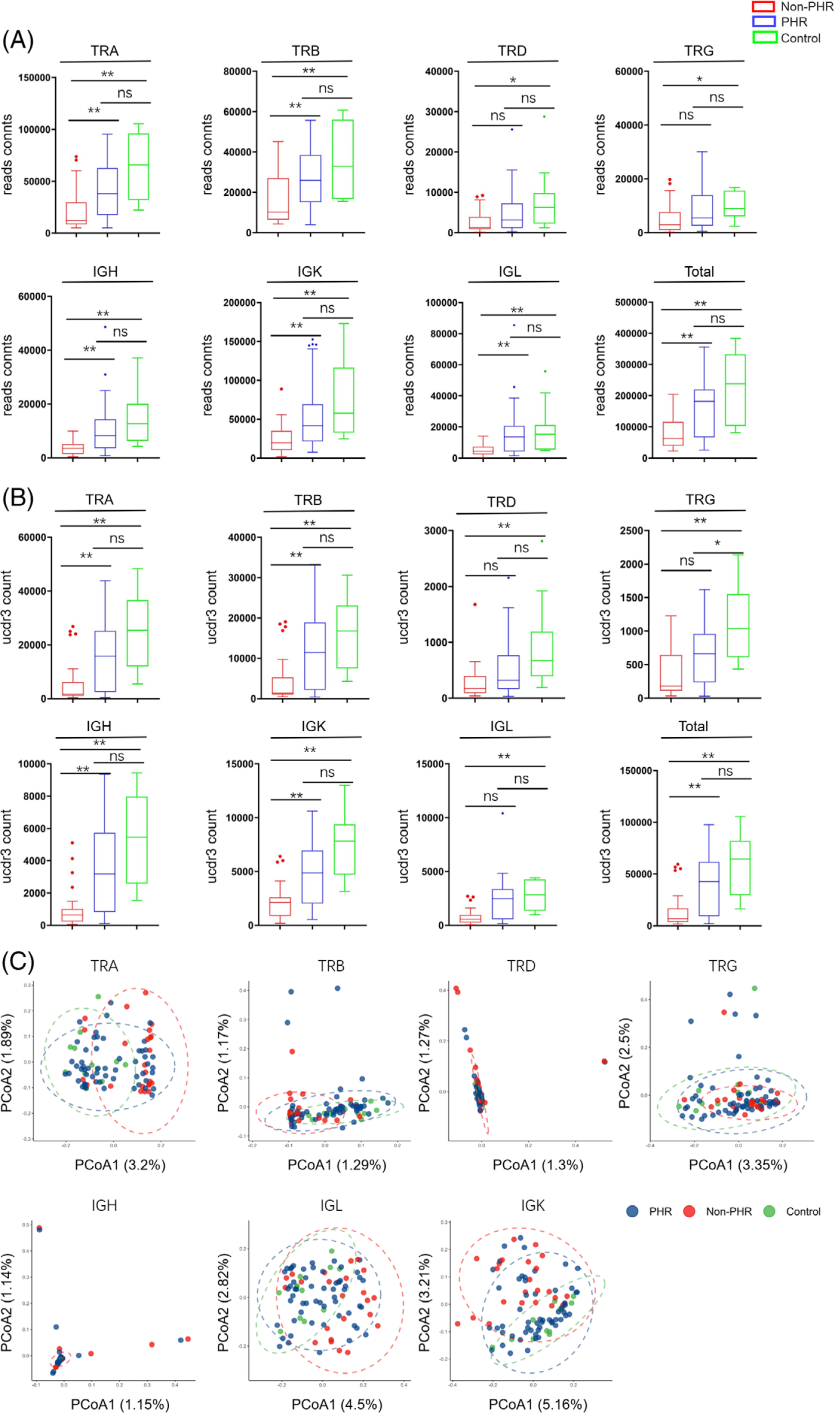
Immune repertoire profile of post‐hospitalization rehabilitation (PHR) and non‐post‐hospitalization rehabilitation (non‐PHR) convalescent COVID‐19 patients. The proportion of reads for overall adaptome immune repertoire and seven‐chain adaptome immune repertoire (A) in healthy control, PHR and non‐PHR subjects. The abundance of immune repertoire measured by the number of unique CDR3 (uCDR3) in the overall adaptome and seven‐chain adaptome (B). Principle of component analysis (PoCA) based on the abundance of TRA, TRB, TRD, TRG, IGH, IGK and IGL clones and the distance between the dots indicates the degree of dissimilarity between samples (C). *p < 0.05, **p < 0.01.

**FIGURE 7:**
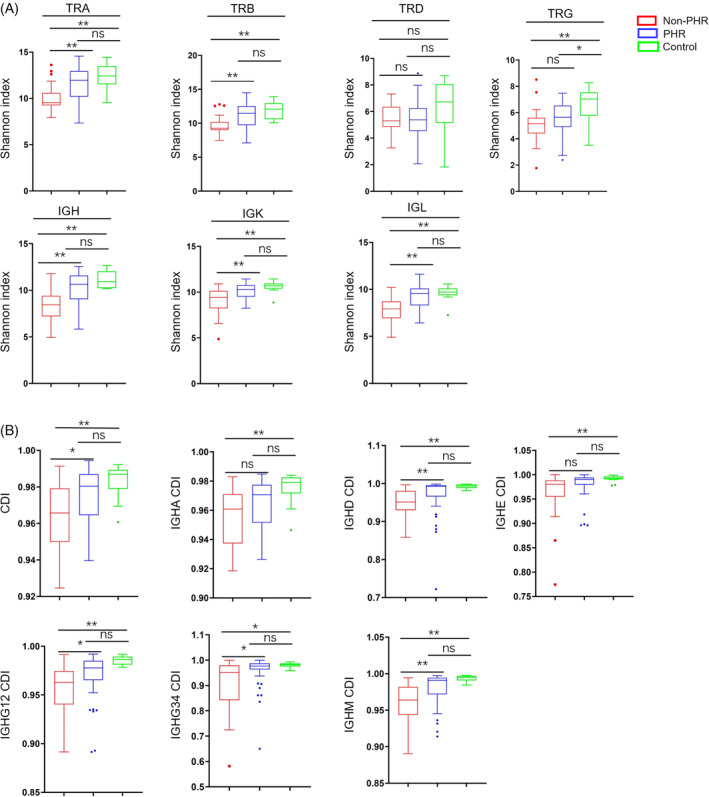
The diversity of adaptome and B cell repertoire clonality in convalescent COVID‐19 patients with or without post‐hospitalization rehabilitation (PHR). The diversity of was demonstrated shannon‐index at the level of unique uCDR3 clones, as shown of TRA, TRB, TCD, TRG, IgH, IgK and IgL (A). Clonal expansion index (CDI) of IGH and IGH isotype (IgHA, IgHD, IgHE, IgHG12, IgHG34 and IgHM) (B).

We further analysed the adaptome immune repertoire of PHR, non‐PHR and healthy control groups. The unique CDR3 count, reflecting the total adaptive immunity, of PHR patients is significantly higher than that of non‐PHR patients. Meanwhile, the PHR uCDR3 count recovered to the level of healthy control (Figure 6A,B, Figure S6), indicating PHR markedly improved adaptive immunity in convalescent patients. In addition, the diversity of the adaptome, defined by the Shannon index, is used to evaluate the potential of the adaptive immune system. Here, we found a significant improvement in the diversity of TRA, TRB, IgH, IgK and IgL for PHR patients compared to non‐PHR patients (Figure 7A). Moreover, the diversity of TRA, TRB, TRG, IgH, IgK and IgL of PHR patients is indistinguishable from healthy controls (Figure 7A).

Meanwhile, the clusters of PHR patients were significantly closer to healthy control clusters compared to non‐PHR patients in TCR (TRA, TRB, TRD and TRG) and BCR (IGH, IGL and IGK) (Figure 6C) in PCoA plots. Furthermore, compared with non‐PHR patients, clonal diversification of IGH, IGHD, IGHM and IGHG were increased in PHR patients (Figure 7B).

**FIGURE 8:**
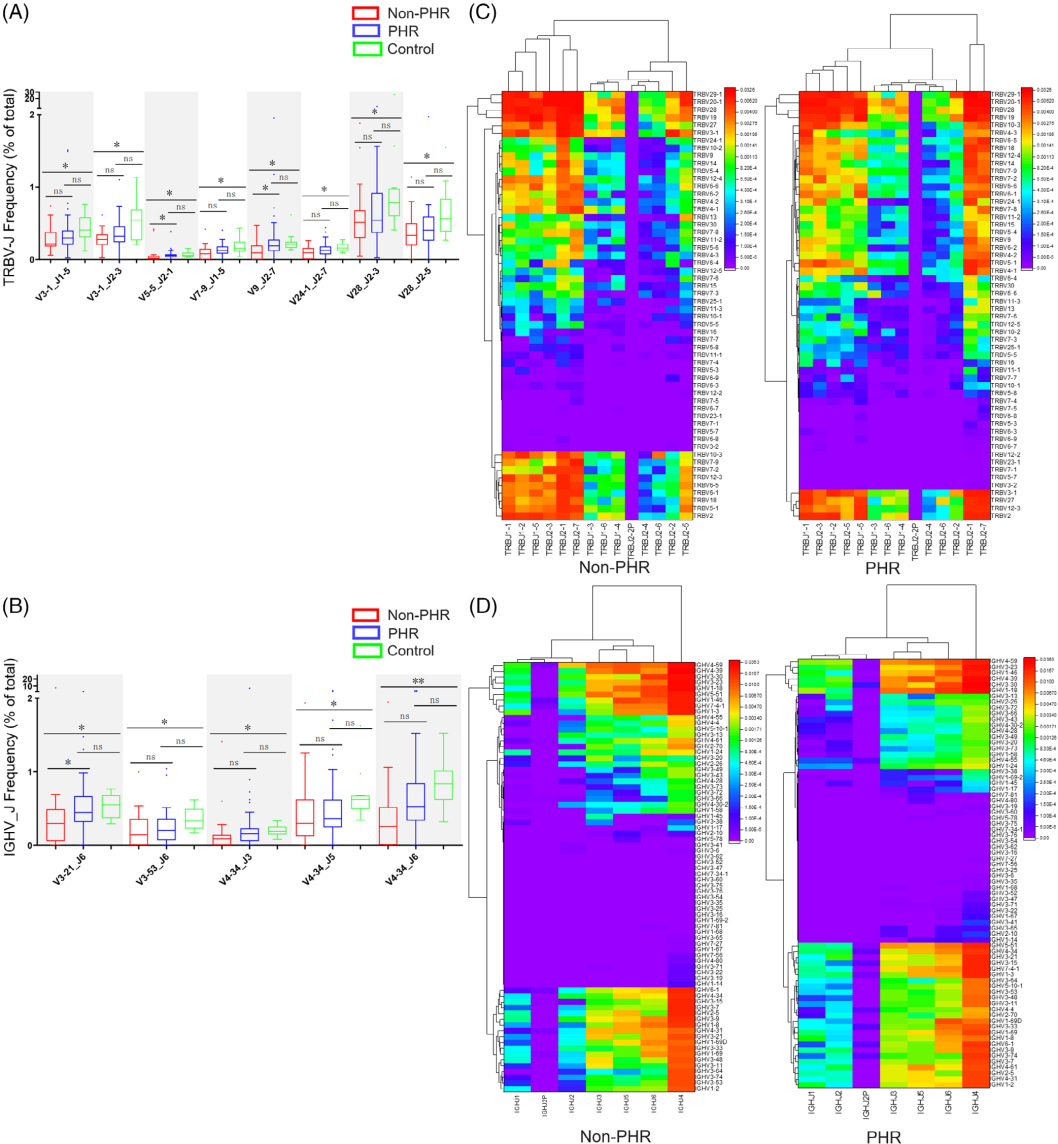
V/J preference usage of post‐hospitalization rehabilitation (PHR) and non‐post‐hospitalization rehabilitation (non‐PHR) convalescent COVID‐19 patients. Kruskal–Wallis test was used to statistically define the significant difference, and data are presented as the mean ± SD values. All of the TRBV‐TRBJs conjunctions (A) and IGHV‐IGHJ conjunctions (B) with *p* value <0.05, between control, PHR and non‐PHR are presented. Heat maps of V gene usage in conjunction with J usage in control, PHR and non‐PHR showing preferred TRBV–TRBJ pairs (C) and IGHV‐IGHJ pairs (D).

In addition, TRBV5‐5_J2‐1, TRBV9_J2‐7 and IGHV3‐21_J6 pairs were significantly down‐regulated in non‐PHR patients compared to healthy controls. Meanwhile, the levels of these pairs were indistinguishable between healthy controls and PHR patients (Figure 8), indicating PHR may correct some skewed V_J pairs caused by COVID‐19 infection. What is more, there was no significant difference in serum cytokine levels between PHR patients, non‐PHR patients and healthy controls (Figure S7).

Hence, although the potency of the adaptive immune system is jeopardized in convalescent patients, PHR can significantly normalize adaptive immune function to a normal state, which validates the necessity of PHR.

## 4 DISCUSSION

We are almost 3 years into COVID‐19 pandemic, different variants of SARS‐COV‐2 has appeared all over the world. Meanwhile, the number of infected subjects might have been largely underestimated, due to the large number of asymptomatic infection and limited testing resources in many areas.^30^, ^31^ Although millions have succumbed to the illness, billions who survived this pandemic have been getting back to normal life. Therefore, it is of great interest and importance to evaluate the potential impact and possible sequela of infection in convalescent COVID‐19 patients. Moreover, as the major mechanism defending against viral infection, including SARS‐CoV‐2 infection,^46^ the condition of immunity, especial adaptive immunity is strongly correlated with the vulnerability, severity, survival and the possibility of multiple infections.^47^, ^48^, ^49^ Therefore, although the major target of SARS‐CoV‐2 infection is lung,^50^, ^51^, ^52^ and multi‐organ damages had been reported upon infection,^53^, ^54^ evaluating long‐term adaptive immune system changes in convalescent COVID‐19 patients remains of great significance. In our study, TCR/BCR immune repertoire profiling reveals the residual alterations in the adaptive immune system of convalescent patients recovering from COVID‐19 6 months after infection, including lymphopenia, reduced diversity, skewed V/J gene usage and B cell repertoire activation. Interestingly, when patients underwent post‐hospitalization rehabilitation after being discharged from hospitals, all of these abnormal patterns could be significantly corrected, which proves the validity and necessity of post‐hospitalization rehabilitation.

Deep sequencing of TCR/BCR adaptome has become a powerful strategy for profiling immune repertoires and reveals clonal selection, expansion and evolution pattern of SARS‐CoV‐2 infection on adaptive immune system.^55^ Seven‐chain immune repertoire profiling was first conducted in a longitudinal holistic for COVID‐19 infection, and demonstrated dynamic changes over the disease course in first month upon infection.^22^ Here, we used the same technology platform to profile the panoramic immune repertoire of long‐term convalescent COVID‐19 patients. Surprisingly, lymphopenia, reduced TCR/BCR diversity remained even six‐month after infection, indicating aberrant immune repertoire after recovery.

A large‐scale, multi‐centre study reported decreased TRB repertoire diversity and skewed TRB V/J utilization in COVID‐19 patients.^56^ Meanwhile, a small‐scale longitudinal study measuring IgH pattern upon COVID‐19 infection yielded no common tendencies among patients, including uCDR3 number, diversity, high‐frequency clone number etc., yet multiple V gene usage preference were discovered in COVID‐19 patients.^57^ In our study, using the holistic immune repertoire technology, we found reduced uCDR3 numbers, reduced repertoire diversity in all seven TCR/BCR chains and skewed TRB and IgH V/J usage in convalescent COVID‐19 patients. In addition, reduced diversity of BCR and TCR in COVID‐19 patients (non‐ICU and ICU) and recovered in convalescent patient was reported.^19^ This finding appears to be not consistent with ours that showing reduced diversity of TCR and BCR, and this discrepancy may cause by the sample number difference. Due to the larger sample size (82 vs. 12) of convalescent COVID‐19 patients in our study, our finding may have more immunological significance.

BCR repertoire is the key factor defining humoral immunity against viral infection, and its feature may serve as a viable predictive biomarker for particular vaccination or actual infection.^58^ Various studies have reported the distinct B cell repertoire pattern in COVID‐19 patients: including distinctive IGHV rearrangements and CDR3H length for B cell heavy chain,^59^ transient IgA surge and characteristic IgM to IgG isotype switch, as well as reduced BCR repertoire diversity during early onset of infection.^22^ Previously, the concept of immune repertoire convergence was used to measure immunogenicity, especially in tumour.^60^ Galson et al. report a stereotypical naive immune response of BCR heavy chain across their study cohort, with clonal expansion and strong convergent signature.^61^ Here, we measured these COVID‐19 specific BCR signature in the convalescent patients, and discovered individual clone expansion and diversification, including somatic hypermutation and class switch. This information supports the potential of BCR signature as a biomarker to measure the recovery of COVID‐19 infection.

Similar to severe acute respiratory syndrome (SARS), middle east respiratory syndrome (MERS) and influenza, T and B lymphocytes also plays the key role in defending against SARS‐CoV‐2 infections.^46^, ^47^ Moreover, the full recovery of adaptive immunity may take more than 2 years in convalescent SARS patients.^48^ Therefore, exploring the immunological characteristics of convalescent COVID‐19 patients will help to understand the impact of SARS‐CoV‐2 infections on adaptive immune responses more comprehensively, and the diversity of TCR and BCR will provide valuable insights into the dynamic change of immune function in convalescent COVID‐19 patients. In our study, we found that the seropositivity rate of IgA, IgM, IgG and neutralizing antibodies of convalescent COVID‐19 patients were 53.75%, 25%, 92.5% and 92.5%, respectively. However, the IgA, IgM, IgG and neutralizing antibodies of 12 healthy volunteers are negative. In addition, decreased T and B cell repertoire diversity also indicates a dysfunctional immune response in convalescent COVID‐19 patients. Taken together, this evidence suggests that convalescent COVID‐19 patients may take a long time to fully recover adaptive immunity, even more than 6 months. Therefore, exploring the dynamic change of immune function in convalescent COVID‐19 patients will be a very worthwhile subject in further.

Multiple evidences are supporting residual multi‐system symptoms and complications after the initial stage of acute infection.^62^ A systematic review showed that at 6 months after infected SARS‐CoV‐2, 54% of COVID‐19 survivors suffered at least one long‐term symptom.^63^ Fatigue, muscle weakness, sleep difficulties, difficulty with memory and concentration were the most commonly persistent symptoms in convalescent COVID‐19 patients.^63^, ^64^ The latest research data suggests that COVID‐19 will enhance the incidence rate of type 2 diabetes and the risk and 1‐year burden of cardiovascular disease.^65^, ^66^, ^67^ Hence, more and more governments and experts begin to pay attention to long‐term symptom of COVID‐19 survivals. The National Institute for Health and Care Excellence (NICE) published a clinical guideline for managing long‐term symptoms of COVID‐19 patients.^68^ The guideline points out that multidisciplinary rehabilitation is critical for managing hospitalized COVID‐19 patients, but when and how to administrate rehabilitation remains to be further investigation. Therefore, establishing an objective standard is crucial for evaluating the effect of rehabilitation and formulating rehabilitation strategies for convalescent COVID‐19 patients.

Various studies have reported that early rehabilitation is essential for minimizing the risk of developing long‐term symptoms and promoting functional restoration for patients, and rehabilitation should be started as early as possible.^69^, ^70^ The early rehabilitation may be personalized and focus on addressing patient‐specific problems, targeting such as pulmonary and cardiac function, etc. A randomized controlled study showed that a 6‐week respiratory rehabilitation can improve respiratory functions, quality of life, and anxiety in convalescent COVID‐19 patients.^71^ In addition, individuals who suffered from COVID‐19 with continuous respiratory symptoms should be given rehabilitation.^70^ Regular exercise training programs have immune‐regulatory and anti‐inflammatory effects.^72^ For example, aerobic exercise not only can relieve anxiety and depression, but also can enhance aerobic capacity, thus improving short‐term immunity, including increasing immune cell function, immunoglobulins level and improving respiratory function in COVID‐19 patients.^73^ A six‐month study for pulmonary rehabilitation among patients hospitalized with COVID‐19 showed that pulmonary rehabilitation can increase exercise capacity, without altering immunity variables.^74^ Our current study discovered that opportune rehabilitation may normalize the impaired immune functions, such as lymphopenia, TCR/BCR diversity, etc. This crucial finding may change our strategy for post‐hospitalization patients. In addition, evaluating adaptive immune system function using immune repertoire sequencing technology may be an objective way to evaluate the efficacy of rehabilitation strategy from molecular immunology prospect, and more data is needed to establish the standard. Therefore, this study will initiate a trend of biomarker discovery for COVID‐19 recovery, and direct further way of COVID‐19 rehabilitation.

Finally, despite these promising results, some questions remain to be addressed, especially from the micro‐prospect of the immune repertoire, that is clone level analysis need to be further performed. SARS‐CoV‐2 associated/specific clones have been identified in TCRβ^56^ and IgH.^75^ It would be enlightening to perform data mining comparing our data set to the existing COVID‐19 specific clones, and find out the T cell and B cell signatures of COVID‐19 infection recovery.

In summary, our study demonstrated a holistic manifestation of crucial adaptive immune system alteration in long‐term convalescent COVID‐19 patients, involving both T cell and B cell repertoire. Moreover, we discovered that post‐hospitalization rehabilitation markedly improved adaptive immune system function in convalescent COVID‐19 patients, which may help avoid the occurrence of immune diseases. Finally, our findings offer a new strategic avenue for guiding COVID‐19 patients back to normal life.

## AUTHOR CONTRIBUTIONS

Bing Feng, Danwen Zheng and Zuqing Su performed experiments with the help of Lipeng Tang, Laijun Yang, Xiaohua Xu, Qian Wang, Qiaoli Lin, Jiajun Hu, Meixuan Lin, Liqun Huang, Xin Zhou, Ying Zhu, Han liu, Song Li, Yanjing Lu, Bin Wu and Rongdong Shi; Bing Feng, Wenjing Pan and Zhe Wang analysed the data; Guangjuan Zheng, Yuntao Liu, Jianwen Guo and Banghan Ding performed the experimental design; Zhe Wang, Bing Feng and Zuqing Su wrote the manuscript with input from all the authors; Zhongde Zhang, Guangjuan Zheng and Yunao Liu designed and supervised the overall study.

## CONFLICT OF INTEREST STATEMENT

The authors declare that they have no conflict of interest.

## Supporting information


**Figure S1.** Significant differences of TRBV (A) and IGHV (B) segment usage in convalescent COVID‐19 patients, all of the segments with *p* value <0.05, between control and convalescent COVID‐19 patients are presented.

**Figure S2.** Comparison of CDR3 length of TRBV (A) and IgHV (B) between healthy control and convalescent COVID‐19 patients.

**Figure S3.** CDI comparison between healthy control and convalescent COVID‐19 patients for IgHA (A), IgHD (B), IgHE (C), IgHG12 (D), IgHG34 (E) and IgHM (F).

**Figure S4.** IgH profile comparison between healthy control and convalescent COVID‐19 patients for (A) IgH transitivity, (B) IgH density, (C) IgH average degree, (D) IgH order, (E) IgH mean cluster size, (F) IgH largest cluster, (G) IgH assortativity coefficient and (H) IgH size.

**Figure S5.** The expression of inflammatory cytokines of convalescent COVID‐19 patients.

**Figure S6.** Expression percentage calculated by reads and uCDR3s in each chain from post‐hospitalization rehabilitation (PHR) and non‐post‐hospitalization rehabilitation (non‐PHR) convalescent COVID‐19 patients and Control. (A)The outside circle represents Control, the middle circle represents PHR, and the inside circle represents non‐PHR. (B) Specific statistical analysis of the percentage of each TCR and BCR chains among non‐PHR, PHR patients and Control by counting reads. (C) Specific statistical analysis of the percentage of each TCR and BCR chains among non‐PHR, PHR patients and Control by counting uCDR3. *p < 0.05, **p < 0.01.

**Figure S7.** The expression of inflammatory cytokines of PHR, non‐PHR and control.

## Data Availability

The data that support the findings of this study are available from the corresponding author upon reasonable request.

## References

[1] Wang D , Hu B , Hu C , et al. Clinical characteristics of 138 hospitalized patients with 2019 novel coronavirus‐infected pneumonia in Wuhan, China. JAMA. 2020;323:1061‐1069. 32031570 10.1001/jama.2020.1585 PMC7042881

[2] Huang C , Huang L , Wang Y , et al. 6‐month consequences of COVID‐19 in patients discharged from hospital: a cohort study. Lancet. 2021;397:220‐232. 33428867 10.1016/S0140-6736(20)32656-8 PMC7833295

[3] Zhang J , Lin H , Ye B , et al. One‐year sustained cellular and humoral immunities of COVID‐19 convalescents. Clin Infect Dis. 2021;75:e1072‐e1081. 10.1093/cid/ciab884 PMC8524303 34609506

[4] Kwong CHT , Mu J , Li S , et al. Reviving chloroquine for anti‐SARS‐CoV‐2 treatment with cucurbit[7]uril‐based supramolecular formulation. Chin Chem Lett. 2021;32:3019‐3022. 33840982 10.1016/j.cclet.2021.04.008 PMC8019245

[5] Iannacone M , Guidotti LG . Immunobiology and pathogenesis of hepatitis B virus infection. Nat Rev Immunol. 2022;22:19‐32. 34002067 10.1038/s41577-021-00549-4

[6] Moss P . The T cell immune response against SARS‐CoV‐2. Nat Immunol. 2022;23:186‐193. 35105982 10.1038/s41590-021-01122-w

[7] Sette A , Crotty S . Adaptive immunity to SARS‐CoV‐2 and COVID‐19. Cell. 2021;184:861‐880. 33497610 10.1016/j.cell.2021.01.007 PMC7803150

[8] Shah K , Al‐Haidari A , Sun J , Kazi JU . T cell receptor (TCR) signaling in health and disease. Signal Transduct Target Ther. 2021;6:412. 34897277 10.1038/s41392-021-00823-w PMC8666445

[9] Young RM , Phelan JD , Wilson WH , Staudt LM . Pathogenic B‐cell receptor signaling in lymphoid malignancies: new insights to improve treatment. Immunol Rev. 2019;291:190‐213. 31402495 10.1111/imr.12792 PMC6693651

[10] Han J , Lotze MT . The adaptome as biomarker for assessing cancer immunity and immunotherapy. Methods Mol Biol. 2020;2055:369‐397. 31502161 10.1007/978-1-4939-9773-2_17

[11] Song C , Pan W , Brown B , et al. Immune repertoire analysis of normal Chinese donors at different ages. Cell Prolif. 2022;55:e13311. 35929064 10.1111/cpr.13311 PMC9628227

[12] Davis MM , Bjorkman PJ . T‐cell antigen receptor genes and T‐cell recognition. Nature. 1988;334:395‐402. 3043226 10.1038/334395a0

[13] Li Z , Woo CJ , Iglesias‐Ussel MD , Ronai D , Scharff MD . The generation of antibody diversity through somatic hypermutation and class switch recombination. Genes Dev. 2004;18:1‐11. 14724175 10.1101/gad.1161904

[14] Zhang T , Tian T , Lin Y . Functionalizing framework nucleic‐acid‐based nanostructures for biomedical application. Adv Mater. 2022;34:e2107820. 34787933 10.1002/adma.202107820

[15] Jackson KJ , Kidd MJ , Wang Y , Collins AM . The shape of the lymphocyte receptor repertoire: lessons from the B cell receptor. Front Immunol. 2013;4:263. 24032032 10.3389/fimmu.2013.00263 PMC3759170

[16] Izraelson M , Nakonechnaya TO , Moltedo B , et al. Comparative analysis of murine T‐cell receptor repertoires. Immunology. 2018;153:133‐144. 29080364 10.1111/imm.12857 PMC5765371

[17] Arstila TP , Casrouge A , Baron V , Even J , Kanellopoulos J , Kourilsky P . Diversity of human alpha beta T cell receptors. Science. 2000;288:1135. 10841721 10.1126/science.288.5469.1135a

[18] Pogorelyy MV , Minervina AA , Chudakov DM , et al. Method for identification of condition‐associated public antigen receptor sequences. Elife. 2018;7:e33050. 29533178 10.7554/eLife.33050 PMC5873893

[19] Zhou Y , Zhang J , Wang D , et al. Profiling of the immune repertoire in COVID‐19 patients with mild, severe, convalescent, or retesting‐positive status. J Autoimmun. 2021;118:102596. 33540371 10.1016/j.jaut.2021.102596 PMC7837046

[20] Tian S , Ji K , Wang M , et al. Distinct BCR repertoires elicited by SARS‐CoV‐2 RBD and S vaccinations in mice. Cell Discov. 2021;7:91. 34620836 10.1038/s41421-021-00331-9 PMC8495183

[21] Montague Z , Lv H , Otwinowski J , et al. Dynamics of B cell repertoires and emergence of cross‐reactive responses in patients with different severities of COVID‐19. Cell Rep. 2021;35:109173. 33991510 10.1016/j.celrep.2021.109173 PMC8106887

[22] Niu X , Li S , Li P , et al. Longitudinal analysis of T and B cell receptor repertoire transcripts reveal dynamic immune response in COVID‐19 patients. Front Immunol. 2020;11:582010. 33117392 10.3389/fimmu.2020.582010 PMC7561365

[23] Zhao XN , You Y , Cui XM , et al. Single‐cell immune profiling reveals distinct immune response in asymptomatic COVID‐19 patients. Signal Transduct Target Ther. 2021;6:342. 34531370 10.1038/s41392-021-00753-7 PMC8443960

[24] Zhang F , Gan R , Zhen Z , et al. Adaptive immune responses to SARS‐CoV‐2 infection in severe versus mild individuals. Signal Transduct Target Ther. 2020;5:156. 32796814 10.1038/s41392-020-00263-y PMC7426596

[25] Unterman A , Sumida TS , Nouri N , et al. Single‐cell multi‐omics reveals dyssynchrony of the innate and adaptive immune system in progressive COVID‐19. Nat Commun. 2022;13:440. 35064122 10.1038/s41467-021-27716-4 PMC8782894

[26] Brynjolfsson SF , Sigurgrimsdottir H , Einarsdottir ED , et al. Detailed multiplex analysis of SARS‐CoV‐2 specific antibodies in COVID‐19 disease. Front Immunol. 2021;12:695230. 34177962 10.3389/fimmu.2021.695230 PMC8222737

[27] Nielsen SCA , Yang F , Jackson KJL , et al. Human B cell clonal expansion and convergent antibody responses to SARS‐CoV‐2. Cell Host Microbe. 2020;28:516‐525.e515. 32941787 10.1016/j.chom.2020.09.002 PMC7470783

[28] Sherina N , Piralla A , du L , et al. Persistence of SARS‐CoV‐2‐specific B and T cell responses in convalescent COVID‐19 patients 6–8 months after the infection. Med (N Y). 2021;2:281‐295.e284. 10.1016/j.medj.2021.02.001 PMC7874960 33589885

[29] Wang Y , Duan F , Zhu Z , et al. Analysis of TCR repertoire by high‐throughput sequencing indicates the feature of T cell immune response after SARS‐CoV‐2 infection. Cells. 2021;11:68. 35011632 10.3390/cells11010068 PMC8750083

[30] Wu Z , McGoogan JM . Characteristics of and important lessons from the coronavirus disease 2019 (COVID‐19) outbreak in China: summary of a report of 72 314 cases from the Chinese Center for Disease Control and Prevention. JAMA. 2020;323:1239‐1242. 32091533 10.1001/jama.2020.2648

[31] Hoogenboom WS , Pham A , Anand H , et al. Clinical characteristics of the first and second COVID‐19 waves in the Bronx, New York: a retrospective cohort study. Lancet Reg Health Am. 2021;3:100041. 34423331 10.1016/j.lana.2021.100041 PMC8367084

[32] Tang G , Huang M , Luo Y , et al. The dynamic immunological parameter landscape in coronavirus disease 2019 patients with different outcomes. Front Immunol. 2021;12:697622. 34777333 10.3389/fimmu.2021.697622 PMC8586656

[33] Wu H , Zhu H , Yuan C , et al. Clinical and immune features of hospitalized pediatric patients with coronavirus disease 2019 (COVID‐19) in Wuhan, China. JAMA Netw Open. 2020;3:e2010895. 32492165 10.1001/jamanetworkopen.2020.10895 PMC7272117

[34] Huang J , Alexey S , Li J , et al. Correction: unique CDR3 epitope targeting by CAR‐T cells is a viable approach for treating T‐cell malignancies. Leukemia. 2019;33:2341. 31097784 10.1038/s41375-019-0484-y

[35] Wang C , Sanders CM , Yang Q , et al. High throughput sequencing reveals a complex pattern of dynamic interrelationships among human T cell subsets. Proc Natl Acad Sci U S A. 2010;107:1518‐1523. 20080641 10.1073/pnas.0913939107 PMC2824416

[36] Bashford‐Rogers RJM , Bergamaschi L , McKinney EF , et al. Analysis of the B cell receptor repertoire in six immune‐mediated diseases. Nature. 2019;574:122‐126. 31554970 10.1038/s41586-019-1595-3 PMC6795535

[37] Wang H , Yan D , Li Y , et al. Clinical and antibody characteristics reveal diverse signatures of severe and non‐severe SARS‐CoV‐2 patients. Infect Dis Poverty. 2022;11:15. 35109926 10.1186/s40249-022-00940-w PMC8809634

[38] Fouladseresht H , Talepoor AG , Eskandari N , et al. Potential immune indicators for predicting the prognosis of COVID‐19 and trauma: similarities and disparities. Front Immunol. 2021;12:785946. 35126355 10.3389/fimmu.2021.785946 PMC8815083

[39] Wang L , Ding Y , Li N , et al. Nanobody‐based polyvinyl alcohol beads as antifouling adsorbents for selective removal of tumor necrosis factor‐α. Chin Chem Lett. 2022;33:2512‐2516.

[40] Fouladseresht H , Doroudchi M , Rokhtabnak N , et al. Predictive monitoring and therapeutic immune biomarkers in the management of clinical complications of COVID‐19. Cytokine Growth Factor Rev. 2021;58:32‐48. 33199179 10.1016/j.cytogfr.2020.10.002 PMC7544568

[41] Lucas C , Wong P , Klein J , et al. Longitudinal analyses reveal immunological misfiring in severe COVID‐19. Nature. 2020;584:463‐469. 32717743 10.1038/s41586-020-2588-y PMC7477538

[42] Mairpady Shambat S , Gómez‐Mejia A , Schweizer TA , et al. Hyperinflammatory environment drives dysfunctional myeloid cell effector response to bacterial challenge in COVID‐19. PLoS Pathog. 2022;18:e1010176. 35007290 10.1371/journal.ppat.1010176 PMC8782468

[43] Mitamura Y , Schulz D , Oro S , et al. Cutaneous and systemic hyperinflammation drives maculopapular drug exanthema in severely ill COVID‐19 patients. Allergy. 2022;77:595‐608. 34157151 10.1111/all.14983 PMC8441838

[44] Bekele Y , Sui Y , Berzofsky JA . IL‐7 in SARS‐CoV‐2 infection and as a potential vaccine adjuvant. Front Immunol. 2021;12:737406. 34603318 10.3389/fimmu.2021.737406 PMC8484798

[45] Wang GL , Gao HX , Wang YL , et al. Serum IP‐10 and IL‐7 levels are associated with disease severity of coronavirus disease 2019. Cytokine. 2021;142:155500. 33810947 10.1016/j.cyto.2021.155500 PMC7973056

[46] Khalil BA , Shakartalla SB , Goel S , et al. Immune profiling of COVID‐19 in correlation with SARS and MERS. Viruses. 2022;14(1):164. 10.3390/v14010164 PMC8778004 35062368

[47] Zhao J , Li K , Wohlford‐Lenane C , et al. Rapid generation of a mouse model for Middle East respiratory syndrome. Proc Natl Acad Sci U S A. 2014;111:4970‐4975. 24599590 10.1073/pnas.1323279111 PMC3977243

[48] Li T , Xie J , He Y , et al. Long‐term persistence of robust antibody and cytotoxic T cell responses in recovered patients infected with SARS coronavirus. PLoS One. 2006;1:e24. 17183651 10.1371/journal.pone.0000024 PMC1762349

[49] Qin X , Xiao L , Li N , et al. Tetrahedral framework nucleic acids‐based delivery of microRNA‐155 inhibits choroidal neovascularization by regulating the polarization of macrophages. Bioactive Mater. 2022;14:134‐144. 10.1016/j.bioactmat.2021.11.031 PMC8892086 35310341

[50] Pelaia C , Tinello C , Vatrella A , De Sarro G , Pelaia G . Lung under attack by COVID‐19‐induced cytokine storm: pathogenic mechanisms and therapeutic implications. Ther Adv Respir Dis. 2020;14:1753466620933508. 32539627 10.1177/1753466620933508 PMC7298425

[51] Zhao Z , Zhao Y , Zhou Y , Wang X , Zhang T , Zuo W . Single‐cell analysis identified lung progenitor cells in COVID‐19 patients. Cell Prolif. 2020;53:e12931. 33094537 10.1111/cpr.12931 PMC7645905

[52] Deng J , Wang R , Huang S , Ding J , Zhou W . Macrophages‐regulating nanomedicines for sepsis therapy. Chin Chem Lett. 2023;34:107588.

[53] Yan Z , Yang M , Lai CL . Long COVID‐19 syndrome: a comprehensive review of its effect on various organ systems and recommendation on rehabilitation plans. Biomedicine. 2021;9:966. 10.3390/biomedicines9080966 PMC8394513 34440170

[54] Wu H , Liao S , Wang Y , et al. Molecular evidence suggesting the persistence of residual SARS‐CoV‐2 and immune responses in the placentas of pregnant patients recovered from COVID‐19. Cell Prolif. 2021;54:e13091. 34291856 10.1111/cpr.13091 PMC8420381

[55] Bieberich F , Vazquez‐Lombardi R , Yermanos A , et al. A single‐cell atlas of lymphocyte adaptive immune repertoires and transcriptomes reveals age‐related differences in convalescent COVID‐19 patients. Front Immunol. 2021;12:701085. 34322127 10.3389/fimmu.2021.701085 PMC8312723

[56] Hou X , Wang G , Fan W , et al. T‐cell receptor repertoires as potential diagnostic markers for patients with COVID‐19. Int J Infect Dis. 2021;113:308‐317. 34688948 10.1016/j.ijid.2021.10.033 PMC8530772

[57] Xiang H , Zhao Y , Li X , et al. Landscapes and dynamic diversifications of B‐cell receptor repertoires in COVID‐19 patients. Hum Immunol. 2022;83:119‐129. 34785098 10.1016/j.humimm.2021.10.007 PMC8566346

[58] Nielsen SCA , Boyd SD . Human adaptive immune receptor repertoire analysis‐past, present, and future. Immunol Rev. 2018;284:9‐23. 29944765 10.1111/imr.12667

[59] Jin X , Zhou W , Luo M , et al. Global characterization of B cell receptor repertoire in COVID‐19 patients by single‐cell V(D)J sequencing. Brief Bioinform. 2021;22:bbab192. 34015809 10.1093/bib/bbab192 PMC8194558

[60] Rosenberg SA , Restifo NP . Adoptive cell transfer as personalized immunotherapy for human cancer. Science. 2015;348:62‐68. 25838374 10.1126/science.aaa4967 PMC6295668

[61] Galson JD , Schaetzle S , Bashford‐Rogers RJM , et al. Deep sequencing of B cell receptor repertoires from COVID‐19 patients reveals strong convergent immune signatures. Front Immunol. 2020;11:605170. 33384691 10.3389/fimmu.2020.605170 PMC7769841

[62] Li J , Lai Y , Li M , et al. Repair of infected bone defect with clindamycin‐tetrahedral DNA nanostructure complex‐loaded 3D bioprinted hybrid scaffold. Chem Eng J. 2022;435:134855.

[63] Groff D , Sun A , Ssentongo AE , et al. Short‐term and long‐term rates of postacute sequelae of SARS‐CoV‐2 infection: a systematic review. JAMA Netw Open. 2021;4:e2128568. 34643720 10.1001/jamanetworkopen.2021.28568 PMC8515212

[64] Huang L , Yao Q , Gu X , et al. 1‐year outcomes in hospital survivors with COVID‐19: a longitudinal cohort study. Lancet. 2021;398:747‐758. 34454673 10.1016/S0140-6736(21)01755-4 PMC8389999

[65] Xie Y , Al‐Aly Z . Risks and burdens of incident diabetes in long COVID: a cohort study. Lancet Diabetes Endocrinol. 2022;10:311‐321. 35325624 10.1016/S2213-8587(22)00044-4 PMC8937253

[66] Shi Q , Zhang X , Jiang F , et al. Clinical characteristics and risk factors for mortality of COVID‐19 patients with diabetes in Wuhan, China: a two‐center, retrospective study. Diabetes Care. 2020;43:1382‐1391. 32409504 10.2337/dc20-0598

[67] Xie Y , Xu E , Bowe B , Al‐Aly Z . Long‐term cardiovascular outcomes of COVID‐19. Nat Med. 2022;28:583‐590. 35132265 10.1038/s41591-022-01689-3 PMC8938267

[68] Venkatesan P . NICE guideline on long COVID. Lancet Respir Med. 2021;9:129. 33453162 10.1016/S2213-2600(21)00031-X PMC7832375

[69] McGroder CF , Zhang D , Choudhury MA , et al. Pulmonary fibrosis 4 months after COVID‐19 is associated with severity of illness and blood leucocyte telomere length. Thorax. 2021;76:1242‐1245. 33927016 10.1136/thoraxjnl-2021-217031 PMC8103561

[70] Funke‐Chambour M , Bridevaux PO , Clarenbach CF , et al. Swiss recommendations for the follow‐up and treatment of pulmonary long COVID. Respiration. 2021;100:826‐841. 34091456 10.1159/000517255 PMC8339046

[71] Liu K , Zhang W , Yang Y , Zhang J , Li Y , Chen Y . Respiratory rehabilitation in elderly patients with COVID‐19: a randomized controlled study. Complement Ther Clin Pract. 2020;39:101166. 32379637 10.1016/j.ctcp.2020.101166 PMC7118596

[72] Gleeson M , Bishop NC , Stensel DJ , Lindley MR , Mastana SS , Nimmo MA . The anti‐inflammatory effects of exercise: mechanisms and implications for the prevention and treatment of disease. Nat Rev Immunol. 2011;11:607‐615. 21818123 10.1038/nri3041

[73] Mohamed AA , Alawna M . Role of increasing the aerobic capacity on improving the function of immune and respiratory systems in patients with coronavirus (COVID‐19): a review. Diabetes Metab Syndr. 2020;14:489‐496. 32388326 10.1016/j.dsx.2020.04.038 PMC7186129

[74] Dun Y , Liu C , Ripley‐Gonzalez JW , et al. Six‐month outcomes and effect of pulmonary rehabilitation among patients hospitalized with COVID‐19: a retrospective cohort study. Ann Med. 2021;53:2099‐2109. 34766857 10.1080/07853890.2021.2001043 PMC8592619

[75] Zhang Y , Yan Q , Luo K , et al. Analysis of B cell receptor repertoires reveals key signatures of the systemic B cell response after SARS‐CoV‐2 infection. J Virol. 2022;96:e0160021. 34878902 10.1128/jvi.01600-21 PMC8865482

