# Supplementary figures and images for: Post‐hospitalization rehabilitation alleviates long‐term immune repertoire alteration in COVID‐19 convalescent patients
Source: Cell Prolif. 2023 Mar 20;56(10):e13450. doi: 10.1111/cpr.13450 (PMC10542649; doi:10.1111/cpr.13450)

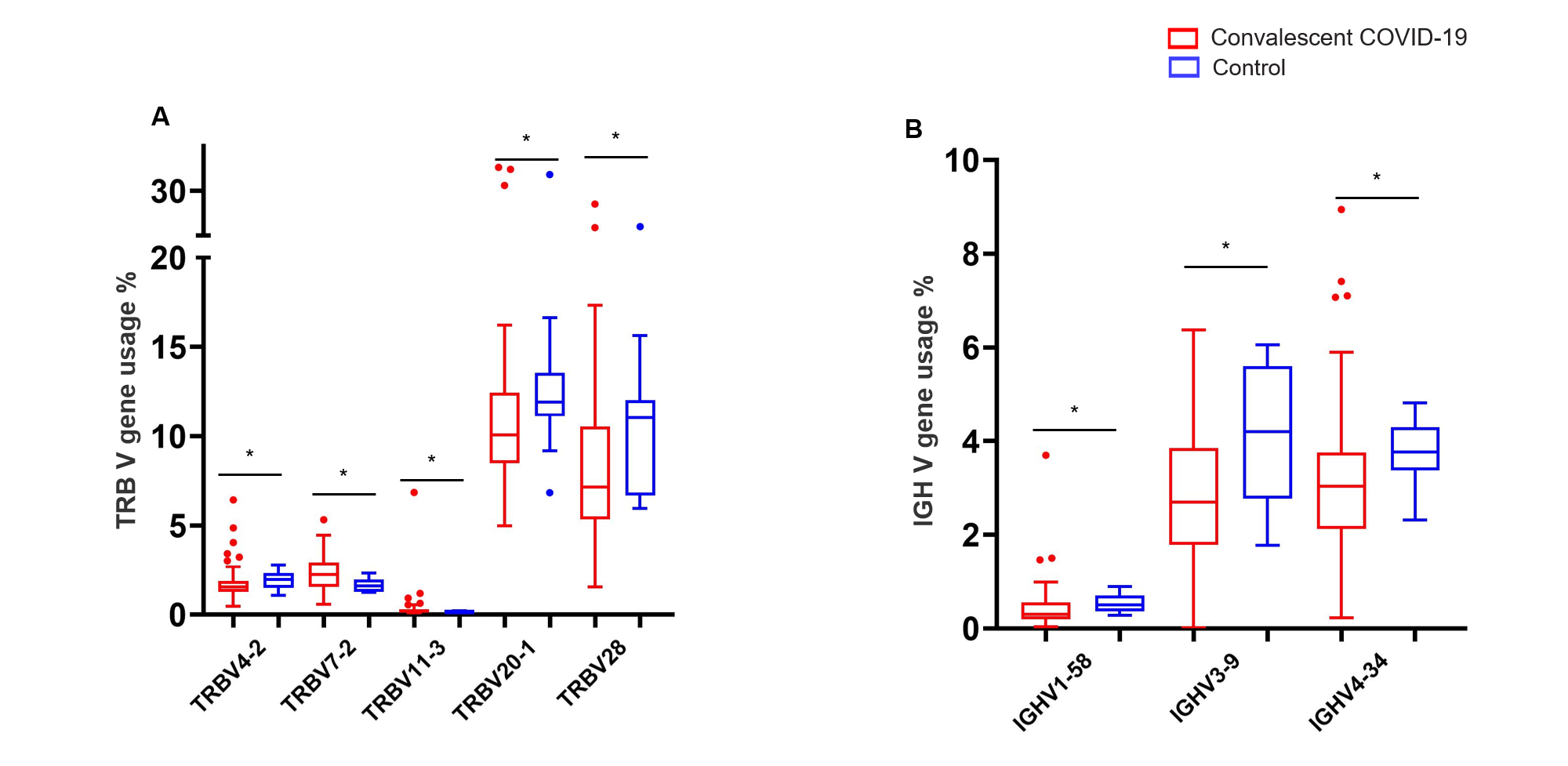

Supplement: Supplementary file 1 — Figure S1. Significant differences of TRBV (A) and IGHV (B) segment usage in convalescent COVID‐19 patients, all of the segments with p value <0.05, between control and convalescent COVID‐19 patients are presented. [file CPR-56-e13450-s006.tif]

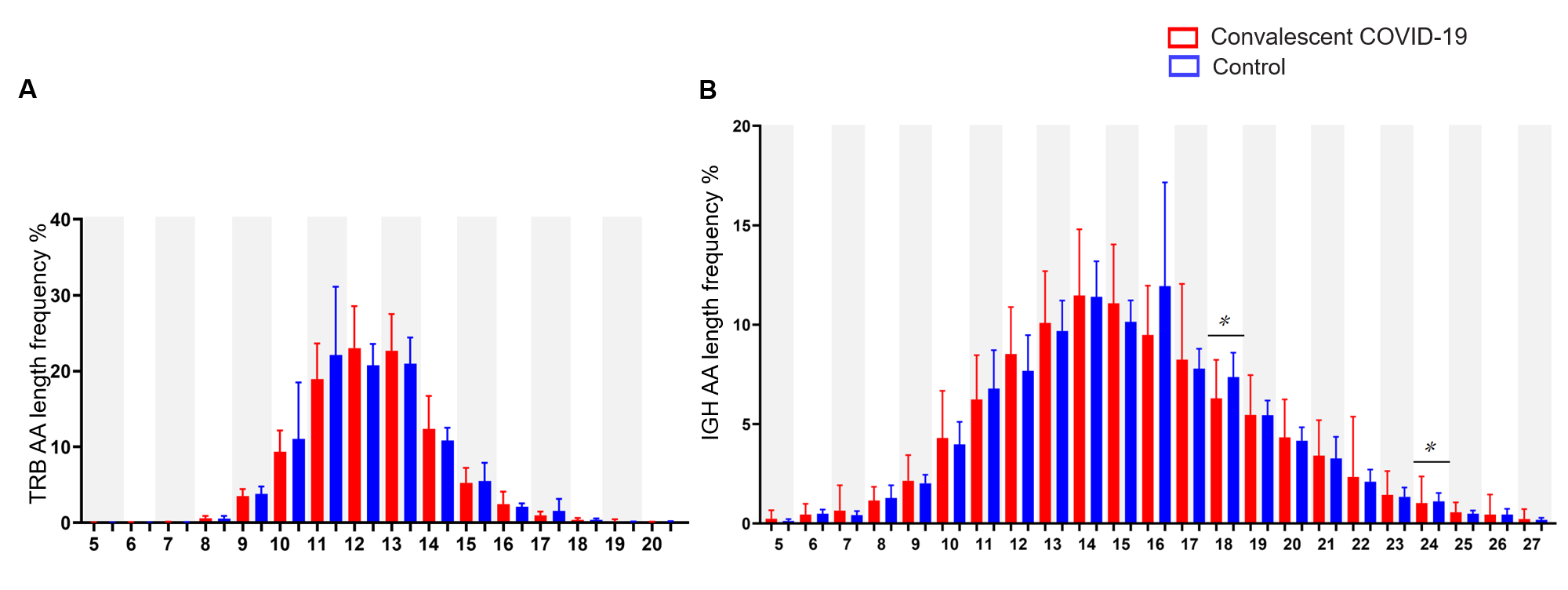

Supplement: Supplementary file 2 — Figure S2. Comparison of CDR3 length of TRBV (A) and IgHV (B) between healthy control and convalescent COVID‐19 patients. [file CPR-56-e13450-s005.tif]

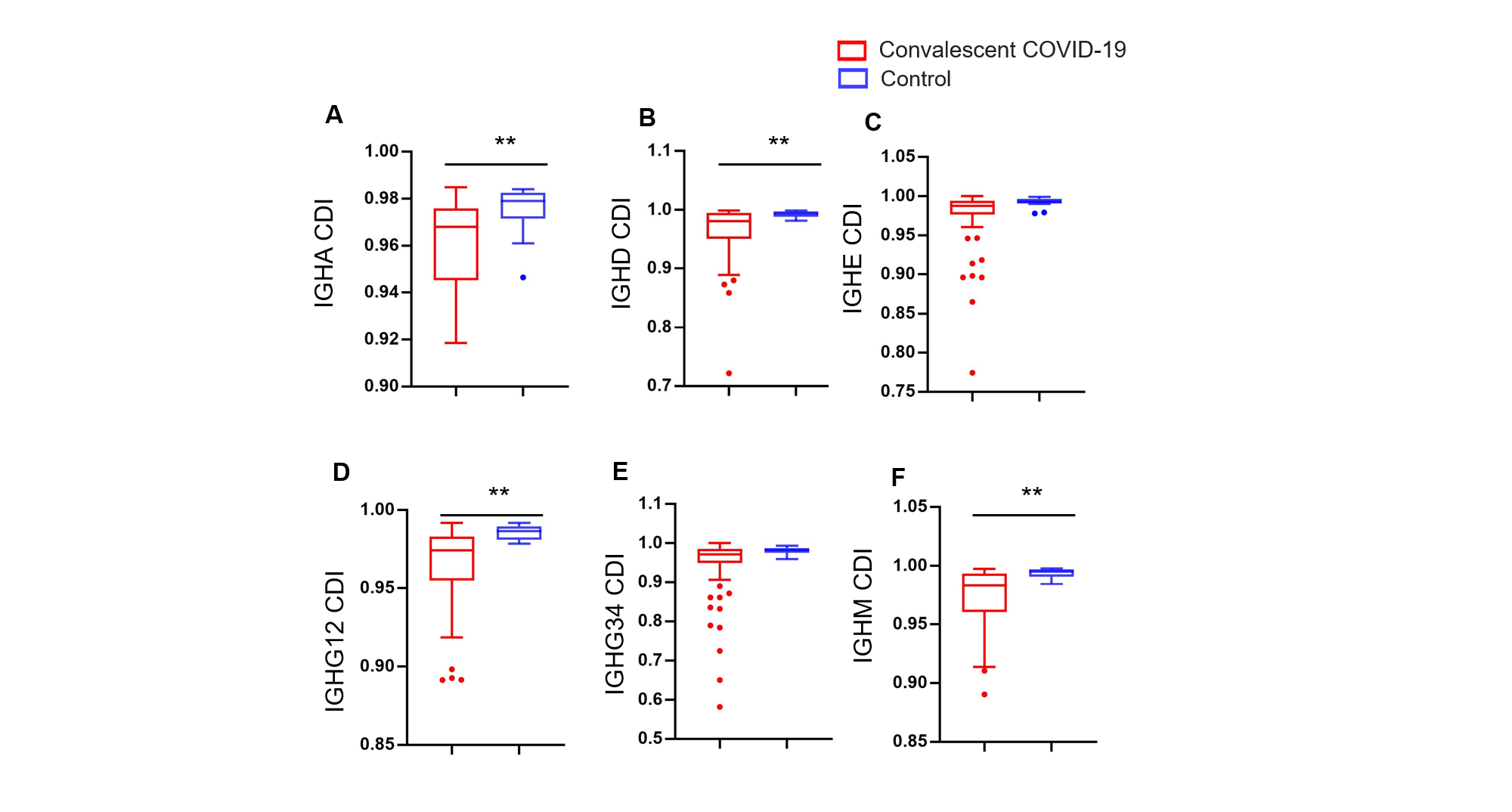

Supplement: Supplementary file 3 — Figure S3. CDI comparison between healthy control and convalescent COVID‐19 patients for IgHA (A), IgHD (B), IgHE (C), IgHG12 (D), IgHG34 (E) and IgHM (F). [file CPR-56-e13450-s001.tif]

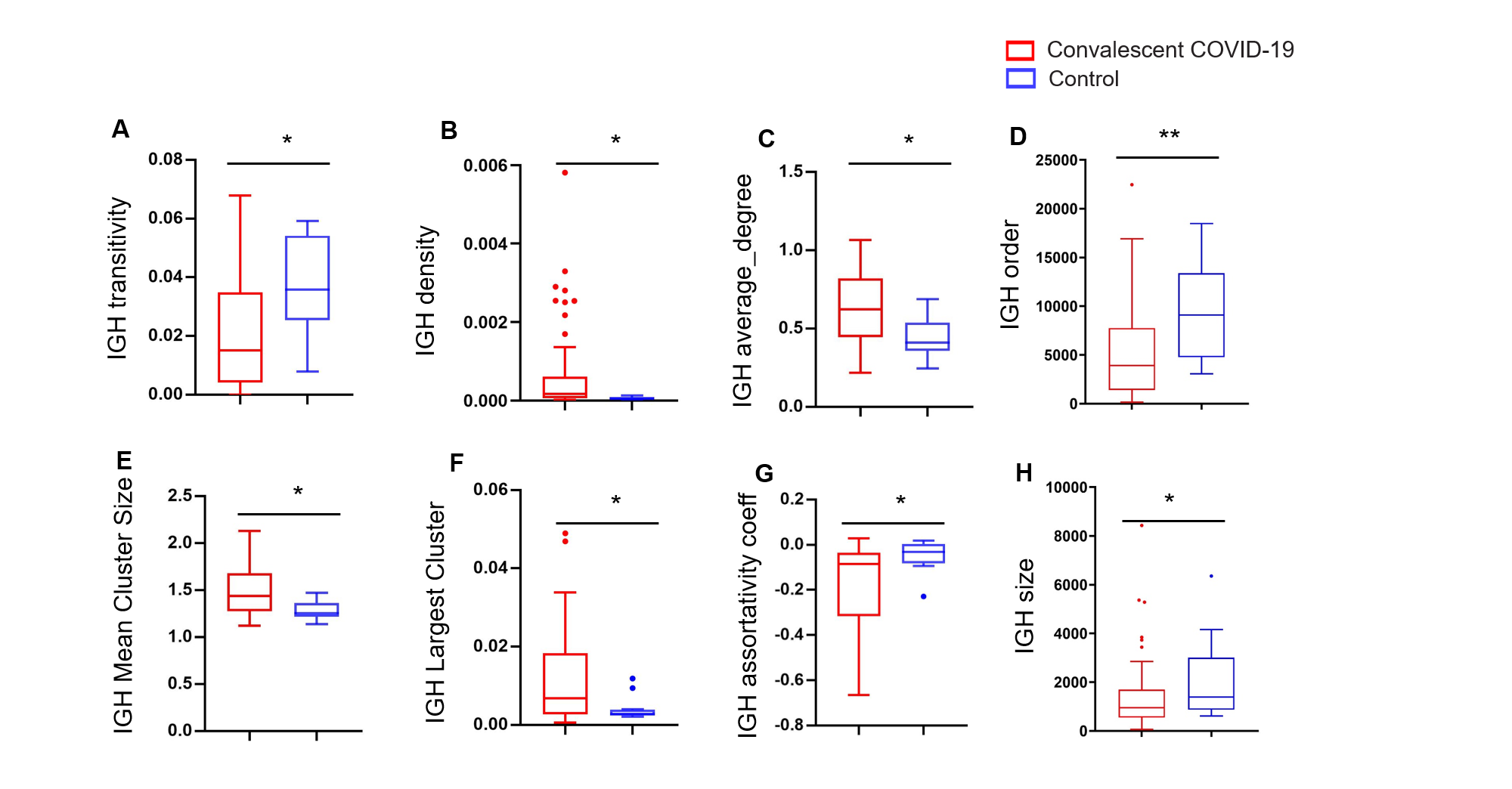

Supplement: Supplementary file 4 — Figure S4. IgH profile comparison between healthy control and convalescent COVID‐19 patients for (A) IgH transitivity, (B) IgH density, (C) IgH average degree, (D) IgH order, (E) IgH mean cluster size, (F) IgH largest cluster, (G) IgH assortativity coefficient and (H) IgH size. [file CPR-56-e13450-s004.tif]

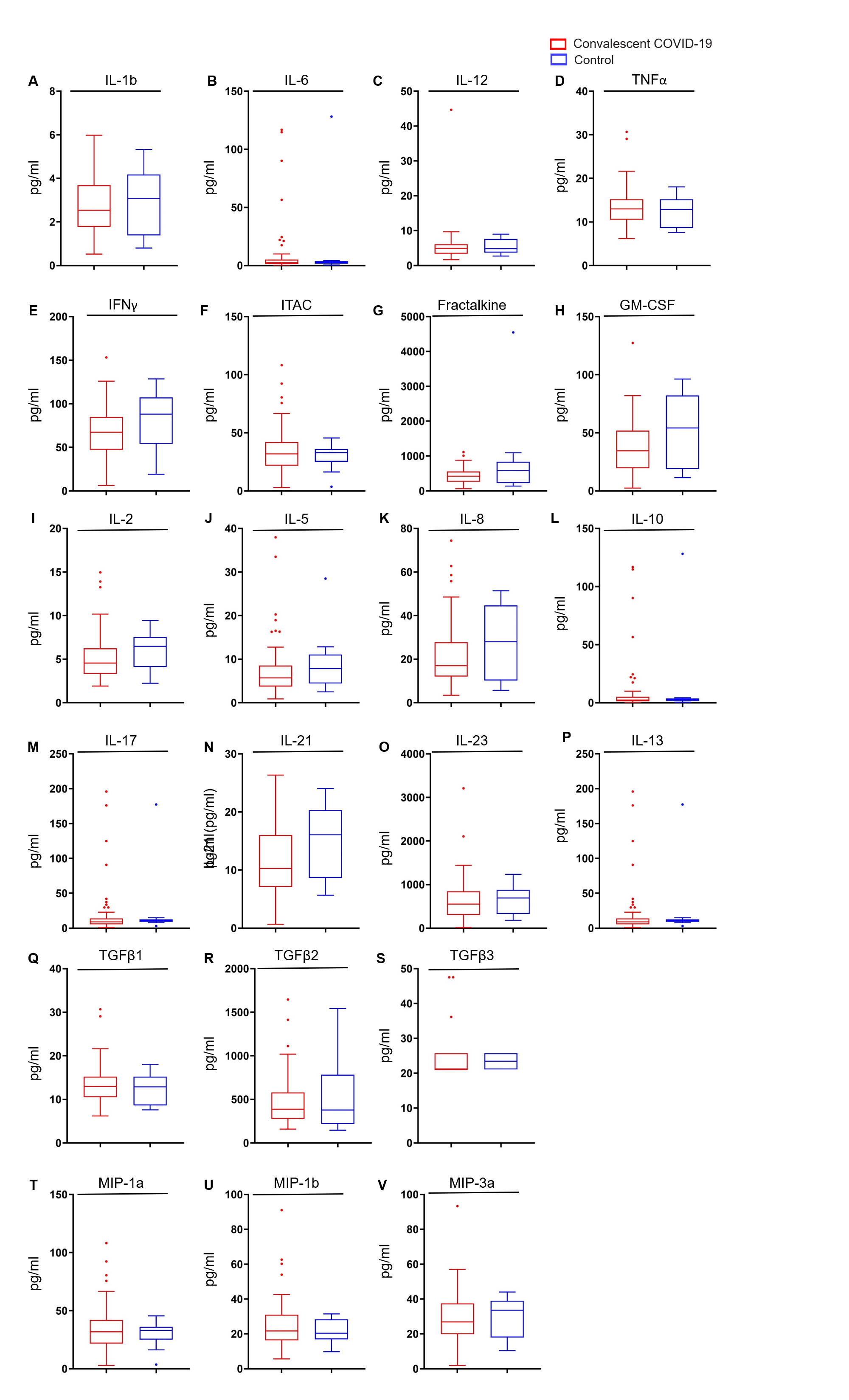

Supplement: Supplementary file 5 — Figure S5. The expression of inflammatory cytokines of convalescent COVID‐19 patients. [file CPR-56-e13450-s007.tif]

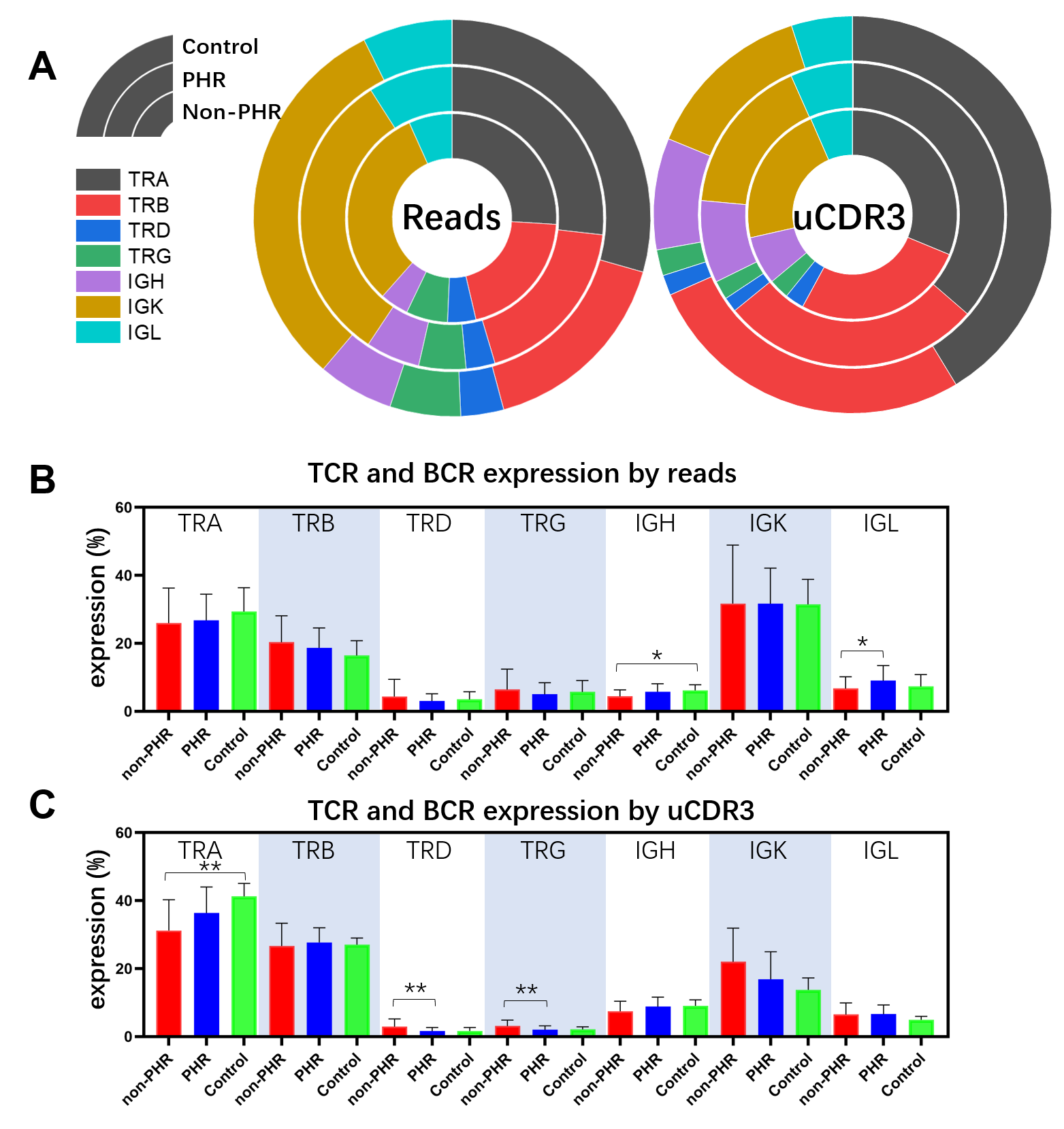

Supplement: Supplementary file 6 — Figure S6. Expression percentage calculated by reads and uCDR3s in each chain from post‐hospitalization rehabilitation (PHR) and non‐post‐hospitalization rehabilitation (non‐PHR) convalescent COVID‐19 patients and Control. (A)The outside circle represents Control, the middle circle represents PHR, and the inside circle represents non‐PHR. (B) Specific statistical analysis of the percentage of each TCR and BCR chains among non‐PHR, PHR patients and Control by counting reads. (C) Specific statistical analysis of the percentage of each TCR and BCR chains among non‐PHR, PHR patients and Control by counting uCDR3. *p < 0.05, **p < 0.01. [file CPR-56-e13450-s002.tif]

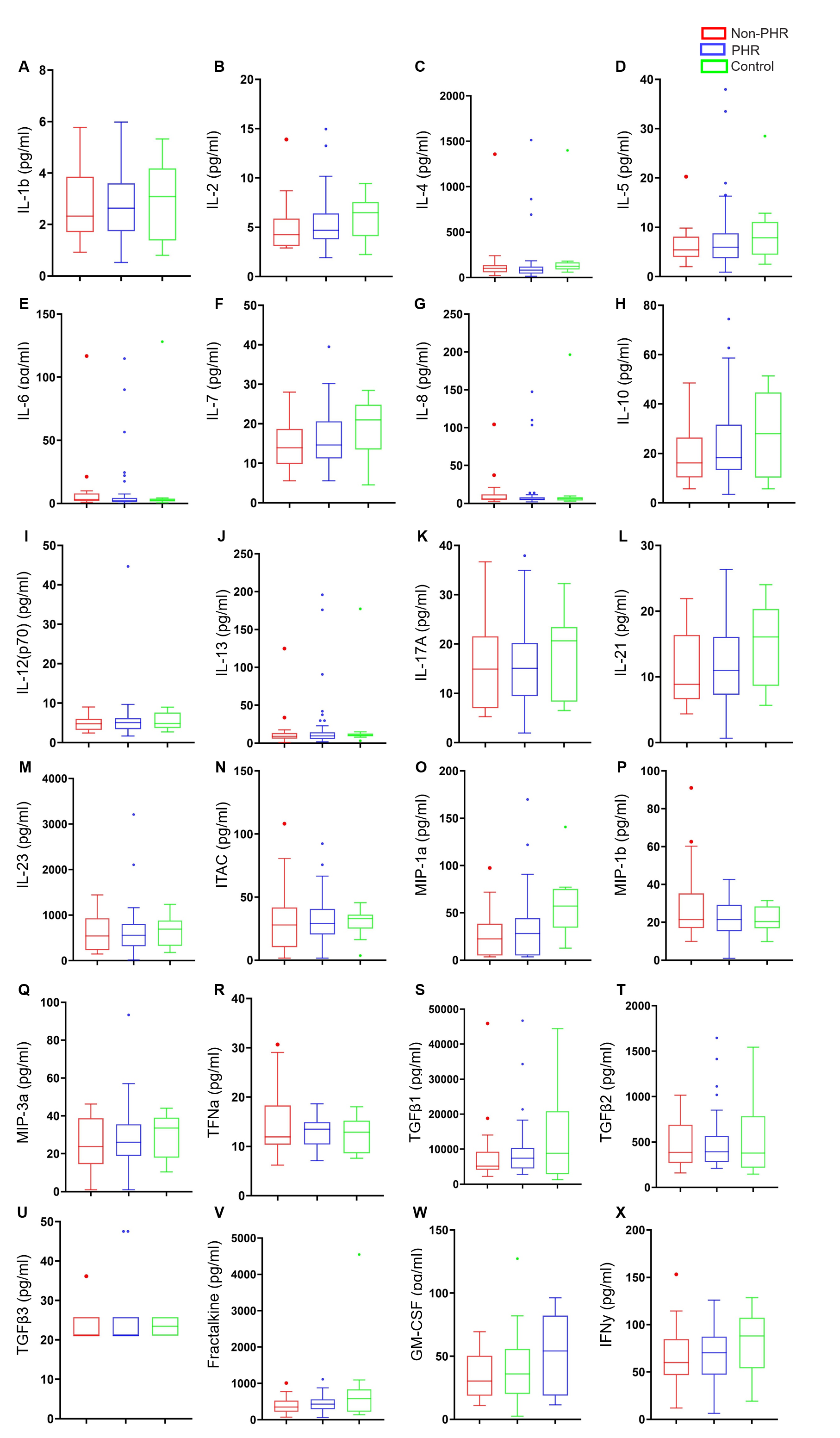

Supplement: Supplementary file 7 — Figure S7. The expression of inflammatory cytokines of PHR, non‐PHR and control. [file CPR-56-e13450-s003.tif]
